# Diagnostic accuracy of AMH for primary ovarian insufficiency/premature ovarian failure: a real-world cohort study

**DOI:** 10.3389/fendo.2026.1742145

**Published:** 2026-02-11

**Authors:** Siyu Mao, Diandian Yang, Xiaoyun Wang, Xiaojing Cao

**Affiliations:** 1 The Second Clinical Medical School, Guangzhou University of Chinese Medicine, Guangzhou, China; 2 The Second Affiliated Hospital, Guangzhou University of Chinese Medicine, Guangzhou, China

**Keywords:** anti-Müllerian hormone (AMH), follicle-stimulating hormone (FSH), ovarian reserve, premature ovarian failure (POF), primary ovarian insufficiency (POI)

## Abstract

**Background/objectives:**

The global incidence of Premature Ovarian Insufficiency (POI)/Primary Ovarian Failure (POF) is rising annually, emerging as a critical health threat affecting women of reproductive age. This condition not only induces menopausal-like symptoms but also leads to severe consequences such as infertility, imposing significant physical, familial, and socioeconomic burdens. Given its detrimental impacts, enhancing preventive strategies is imperative. Anti-Müllerian Hormone (AMH), closely associated with ovarian reserve, has gained increasing application in gynecological diagnostics, particularly exhibiting considerable potential in POI/POF evaluation. However, the diagnostic reliability of AMH remains to be fully elucidated. This study aims to rigorously evaluate the diagnostic accuracy of AMH in POI/POF diagnosis, with the goal of improving diagnostic accuracy to facilitate early detection and intervention, thereby providing evidence-based support for POI/POF prevention efforts.

**Methods:**

A retrospective analysis was conducted on clinical data from 1,106 reproductive-aged women, divided into three groups: DOR group (n=502), POI group (n=143), and POF group (n=461). Statistical analyses were performed using SPSS 26.0. Comparisons of age, AMH, estradiol (E2), prolactin (PRL), luteinizing hormone (LH), follicle-stimulating hormone (FSH), and progesterone (PRG) were conducted. Significant variables (P<0.05) were included in binary logistic regression models. Receiver operating characteristic (ROC) curves evaluated the diagnostic performance of AMH and its combinations with other parameters.

**Results:**

Age, AMH, E2, PRL, LH, FSH, and PRG differed significantly among groups (P<0.05). For POI, the AUC values for individual markers and their combinations were age (0.494), AMH (0.885), E2(0.757), Age+ AMH (0.888), Age+ E2(0.756), AMH+ E2(0.909), Age+ AMH+ E2(0.910). For POF, the AUC values for individual markers and their combinations were age (0.526), AMH (0.872), E2 (0.803), Age+ AMH (0.877), Age+ E2 (0.805), AMH+ E2 (0.910), Age+ AMH+ E2 (0.912).

**Conclusions:**

AMH demonstrates robust diagnostic accuracy for POI/POF diagnosis, with significantly lower levels in affected patients compared to healthy controls. Its diagnostic performance surpasses age and E2, particularly for POF, supporting its utility as a standalone biomarker. These findings advocate for integrating AMH measurement into clinical protocols for early ovarian dysfunction detection in reproductive-aged women.

## 1 Introduction

Ovarian function plays a pivotal role throughout a woman’s life. Normal ovarian hormones maintain regular menstrual cycles and reproductive capacity. Both endogenous and exogenous factors can lead to diminished ovarian function. The decline in female fertility associated with advancing age is attributed to the reduction in both quantity and quality of oocytes, a natural physiological process termed diminished ovarian reserve (DOR). Premature ovarian insufficiency (POI) is characterized by menopausal symptoms occurring before the age of 40, representing early ovarian dysfunction. According to current guidelines, the diagnostic criteria include menstrual irregularities (either failure to establish regular cycles or amenorrhea for ≥4 months) and two elevated follicle-stimulating hormone (FSH) levels (>25 U/L) measured at least 4 weeks apart (1–3). Without intervention, ovarian function may further decline to premature ovarian failure (POF), the terminal stage of ovarian dysfunction occurring before age 40 (4–6).

With the advancement of society and the acceleration of the pace of life, women are increasingly affected by ovarian dysfunction due to various factors such as stress and lifestyle. Epidemiological studies have indicated that the global prevalence of POI ranges from 1% to 3.7% (7–9), with significant variations observed across different age groups and ethnic populations (7, 10). Moreover, in recent years, there has been a noticeable upward trend in the incidence of POI/POF on a global scale. Ovarian failure has a profound impact on hormone secretion, particularly sex hormones. As a result, women may experience a range of menopausal - like symptoms, including hot flashes, irritability, and insomnia (11, 12). Furthermore, the risk of developing cardiovascular diseases, dyslipidemia, and glucose metabolism disorders is significantly elevated (7, 13). Another critical issue arising from declining ovarian reserve is infertility (14, 15). The normal production, development, and release of follicles are prerequisites for fertility. However, a decrease in ovarian reserve can impair the ability to produce mature follicles in multiple ways, thereby affecting a woman’s reproductive capacity. In conclusion, POI/POF represents a significant health challenge for women of reproductive age. It not only has a profound impact on individual well - being but also poses substantial social and public health implications. Given its profound public health implications, strengthening preventive strategies has become imperative.

As we all know, the ovarian follicle pool is finite in females, with follicle numbers steadily decreasing—rather than replenishing—after menarche due to monthly ovulation. The exhaustion of ovarian follicular reserve ultimately leads to menopause. POI or POF occurs when this process accelerates or manifests prematurely. Follicular development is intricately regulated by hormonal signaling. Under the influence of cyclical endocrine fluctuations, growing follicles progress through stages of maturation until a dominant follicle is selected for ovulation. FSH, a glycoprotein hormone synthesized and secreted by pituitary gonadotrope cells, plays a pivotal role in this process. As an anterior pituitary hormone, FSH stimulates granulosa cell proliferation and differentiation, thereby promoting follicular maturation and ovarian growth (16–19). Consequently, serum FSH levels serve as a key indicator of ovarian reserve; elevated FSH concentrations typically reflect compensatory pituitary activity in response to diminishing ovarian function (18). In current clinical practice, FSH measurement remains the sole diagnostic criterion for POI/POF (3, 20). However, its utility is constrained by physiological variability—FSH levels fluctuate significantly across the menstrual cycle, with the early follicular phase (days 2–4 of menstruation) representing the optimal diagnostic window. For patients with POI/POF, who are often present with irregular menses or amenorrhea, timely FSH assessment during the follicular phase becomes challenging, leading to delayed diagnosis and suboptimal disease management (21).

Early identification of POI and POF is critical for women’s reproductive health management. However, conventional diagnostic approaches relying on menstrual history evaluation and cyclic hormone testing suffer from limitations such as poor timeliness and low accessibility (22). Anti-Müllerian hormone (AMH), a member of the TGF-β superfamily primarily secreted by granulosa cells of growing follicles (23), plays a pivotal role in regulating early folliculogenesis and protecting follicles from atresia, thereby serving as a direct biomarker of ovarian reserve (24, 25). As a cycle-independent and easily measurable indicator, AMH offers a potential tool for high-risk population screening at community or primary care levels. Validating its clinical efficacy could facilitate the establishment of a cost-effective, widely accessible early warning system, enabling timely referral to specialized care and reducing long-term risks of infertility, osteoporosis, and associated public health burdens.

While the biological rationale for AMH in predicting POI is well-established (26), existing evidence predominantly derives from small clinical cohorts or specific subgroups, with limited large-scale real-world longitudinal validation. The diagnostic reliability of AMH as an independent predictor in general populations, including threshold determination and prognostic relevance, remains inadequately characterized. This study aims to systematically evaluate the diagnostic accuracy of AMH for POI/POF and define its diagnostic thresholds across populations. By addressing these evidence gaps, the research seeks to inform the integration of AMH into routine screening guidelines, advancing reproductive health management from reactive care to proactive prevention and optimizing public health resource allocation.

## 2 Materials and methods

### 2.1 Study population

After reviewing the medical records of patients meeting the following inclusion criteria and applying both exclusion criteria and diagnostic criteria for selection, a total of 1,106 patients were ultimately included in the study. A total of 1,106 patients were enrolled from Guangdong Provincial Hospital of Chinese Medicine between January 2012 and April 2025. All participants underwent assessment of anti-Müllerian hormone (AMH) levels and six sex hormone parameters (follicle-stimulating hormone (FSH), estradiol (E2), luteinizing hormone (LH), prolactin (PRL), progesterone (PRG), and testosterone (TSTO)). The study cohort was determined following application of inclusion and exclusion criteria. The Medical Ethics Committee of Guangdong Provincial Hospital of Traditional Chinese Medicine reviewed and approved the study protocol (Approval No.: ZE2025-004-01).

#### 2.1.1 Inclusion criteria

(1) Age between 14 and 40 years (inclusive); (2) FSH level > 10 U/L; (3) Availability of complete clinical data.

#### 2.1.2 Exclusion criteria

Patients were excluded if they met any of the following:

(1) Chronic use of medications affecting sex hormone levels (e.g., desogestrel/ethinylestradiol, glucocorticoids); (2) Presence of comorbidities influencing sex hormone regulation (e.g., polycystic ovary syndrome (PCOS), prolactinoma); (3) Active infection, hematological disorders, or related diagnoses; (4) History of malignancy (e.g., breast cancer); (5) Severe hepatic or renal dysfunction.

#### 2.1.3 Diagnostic criteria for POI, POF and DOR

According to the diagnostic standards established by ESHRE, American Society for Reproductive Medicine (ASRM), Centre for Research Excellence in Women’s Health in Reproduction Life (CRE-WHiRL) and International Menopause Society (IMS), elevated FSH levels serve as key diagnostic indicators. The diagnostic criteria are as follows:

a. Diagnostic Criteria for Primary Ovarian Insufficiency (POI) (20)

(1) Two consecutive serum FSH measurements >25 IU/L, obtained at least 4 weeks apart.
(2) Menstrual irregularity defined as: Failure to establish regular menstrual cycles, or Amenorrhea for ≥4 months.

b. Diagnostic Criteria for Premature Ovarian Failure (POF) (4)

(1) Two consecutive serum FSH measurements >40 IU/L, obtained at least 4 weeks apart.
(2) Menstrual irregularity defined as: Failure to establish regular menstrual cycles, or Amenorrhea for ≥4 months.

c. Diagnostic Criteria for Decreased Ovarian Reserve (DOR) (4, 27)

(1) Two consecutive serum FSH measurements >10 IU/L, obtained at least 4 weeks apart.
(2) With or without menstrual irregularities.

### 2.2 Hormone measurement

Peripheral blood sex hormone six-item testing (E2, PRL, LH, FSH, TSTO, and PRG) and AMH detection were performed at Guangdong Provincial Hospital of Traditional Chinese Medicine during days 2–4 of the menstrual cycle or at random for women with infrequent or prolonged amenorrhea. All blood indices mentioned above were measured using electrochemiluminescence immunoassay (ECLIA) throughout the study. Consistent use of the same batch of analytical instruments and methods was maintained across all assays. Quality control certificates from the randomly selected hospital laboratory and instruction manuals for AMH quality control materials are included in the **Supplementary Materials**.

### 2.3 Research methodology

A retrospective cohort analysis was conducted on laboratory parameters obtained from 1,106 patients. The study focused on AMH levels and sex hormone profiles, including FSH, E2, LH, PRL, PRG, and TSTO. All assays were performed at Guangdong Provincial Hospital of Chinese Medicine.

### 2.4 Statistical analyses

SPSS 26.0 statistical software was used for the statistical analyses, with α= 0.05 as the test level of significance and p<0.05 considered statistically significant. For the missing values and values below the detection limit in the dataset, apply multiple imputation to supplement them before proceeding with analysis. For continuous variables that follow a normal distribution, one-way analysis of variance (ANOVA) is employed, with results expressed as mean ± standard deviation (x ± s). For continuous variables that do not conform to a normal distribution, non-parametric tests (specifically the Kruskal- Wallis test) are utilized, and results are presented as median (P25, P75). Binary logistic regression and Box-Tidwell test were used to analyze the correlation between age/AMH/E2 and POI/POF in the reproductive-aged women, and subject work characteristic (ROC) curves were plotted and the lower part of the curves and the critical values were calculated. When conducting ROC analysis, if inconsistent curve directions occur, correct the data before proceeding with the analysis.

## 3 Results

**Figure 1:**
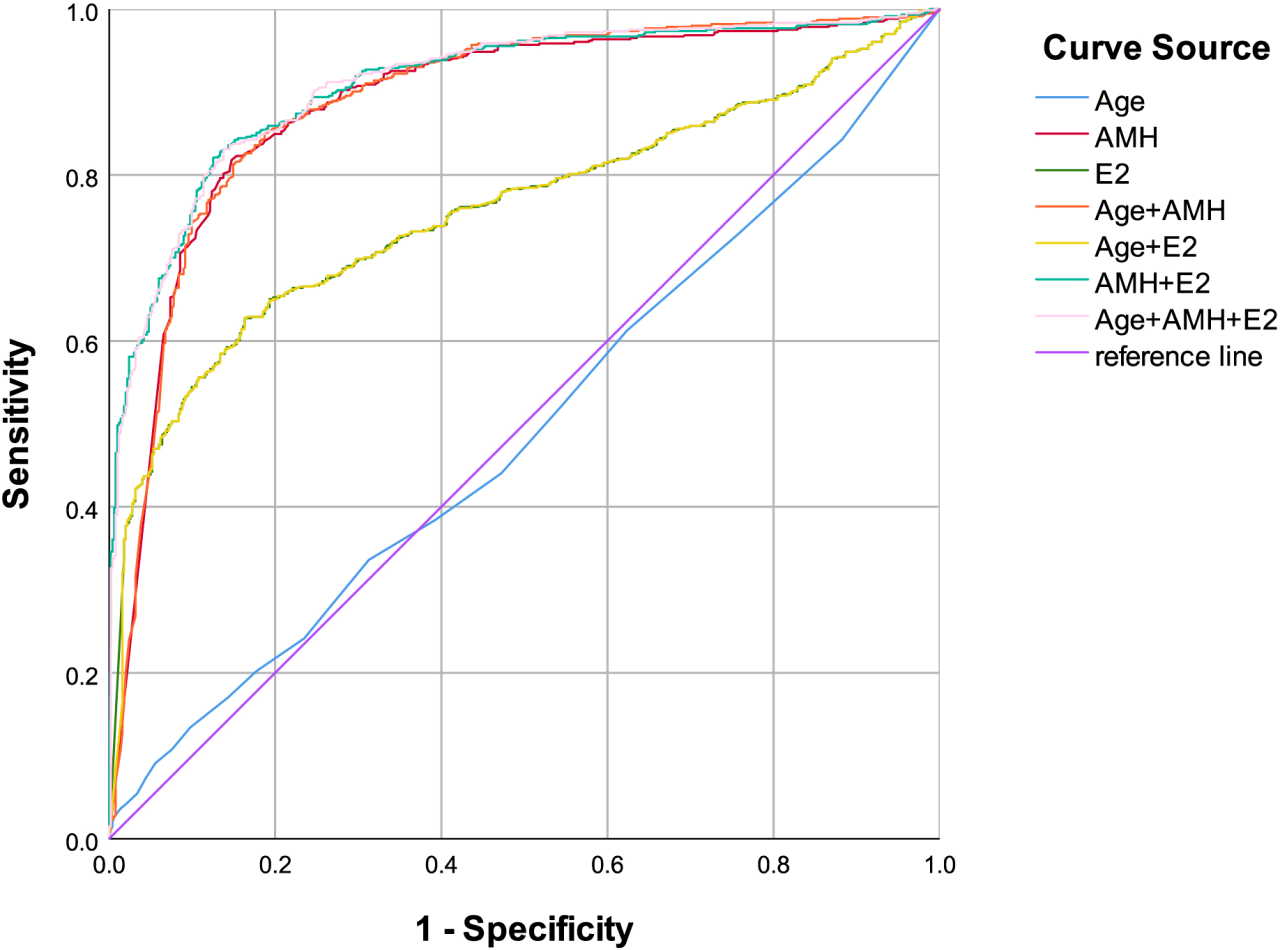
Age, AMH, E2, combination of Age and AMH, combination of Age and E2, combination of AMH and E2, combination of Age, AMH and E2 as ROC curves for the development of POI in the reproductive-aged women.

**Figure 2:**
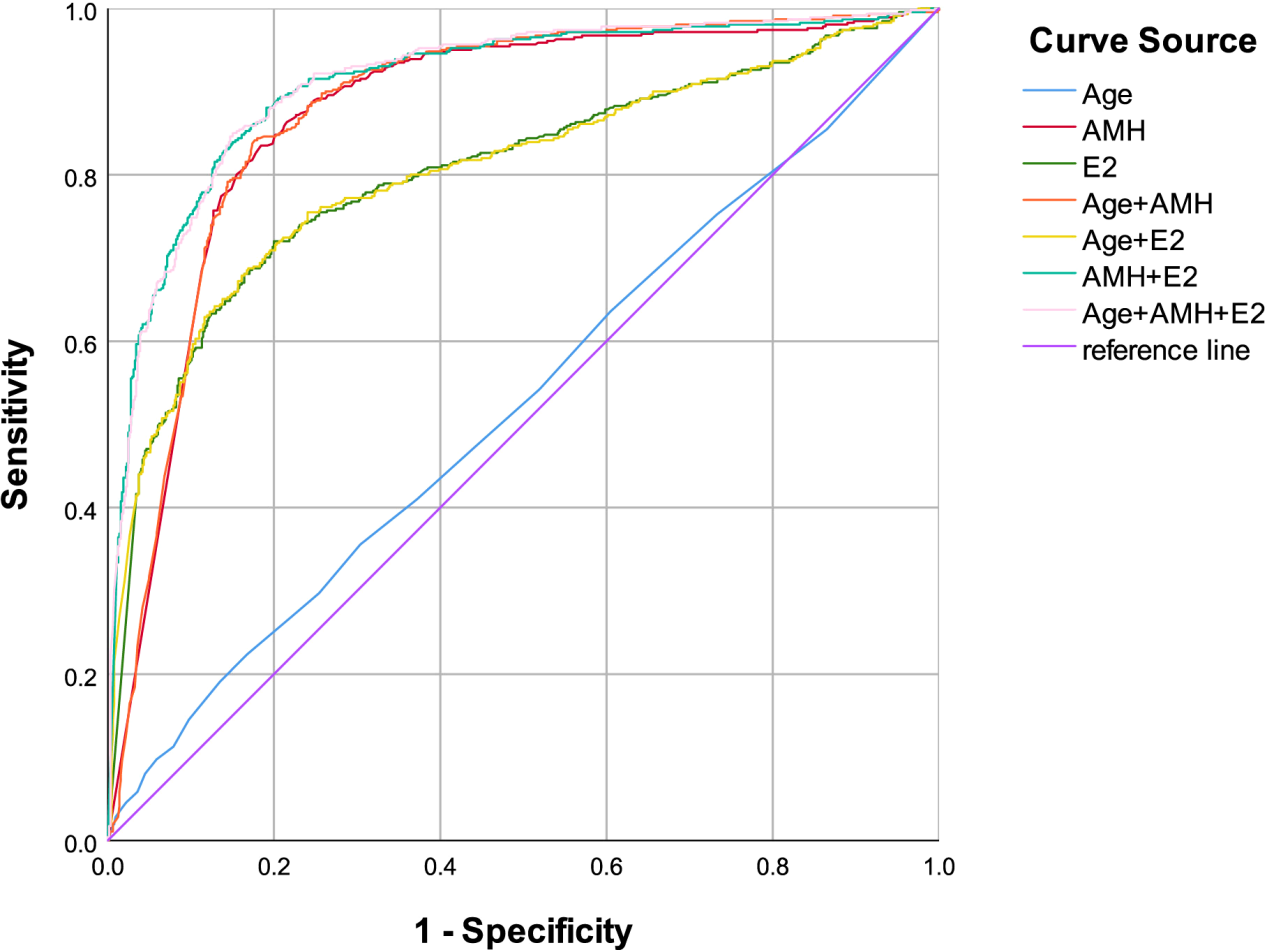
Age, AMH, E2, combination of Age and AMH, combination of Age and E2, combination of AMH and E2, combination of Age, AMH and E2 as ROC curves for the development of POF in the reproductive-aged women.

This study enrolled 1,106 reproductive-aged women aged 16–40 years (mean age ± standard deviation: 34.73 ± 4.88 years), categorized into three groups: 502 cases in the DOR group, 143 cases in the POI group, and 461 cases in the POF group. Comparative analysis of baseline demographic and clinical parameters revealed statistically significant differences among the three groups in age, AMH, E2, PRL, LH, FSH, and PRG levels (see **Table 1** for detailed comparisons). Prior studies and clinical guidelines have consistently identified E2 and FSH as established serum biomarkers for POI/POF diagnosis (21). Given the well-documented age-related decline in ovarian function, our analysis prioritized these variables alongside AMH. Binary logistic regression and Box-Tidwell test were employed to evaluate associations between age, E2, AMH, and the risk of POI/POF (see **Tables 2**, **3**). Receiver operating characteristic (ROC) curve analyses were subsequently performed to assess the diagnostic performance of these markers. For POI, the AUC values for individual markers and their combinations were as follows: age (0.494), AMH (0.885), E2(0.757), Age+ AMH (0.888), Age+ E2(0.756), AMH+ E2(0.909), Age+ AMH+ E2(0.910) (see **Figure 1**, **Table 4**). For POF, the AUC values for individual markers and their combinations were as follows: Age (0.526), AMH (0.872), E2 (0.803), Age+ AMH (0.877), Age+ E2 (0.805), AMH+ E2 (0.910), Age+ AMH+ E2 (0.912) (see **Figure 2**, **Table 5**).

**Table 1:** Comparison of general information in reproductive-aged women.

Characteristics	DOR group (n = 502)	POI group (n = 143)	POF group (n = 461)	Between-group P
Age	34.84 ± 4.50	35.57 ± 4.85	34.34 ± 5.25	**0.018**
AMH (ng/mL)	0.293(0.079,0.620)	0.029(0.005,0.101)	0.005(0.001,0.014)	**<0.001**
E2 (pmoL/L)	146.05(96.03,239.70)	127.61(56.45,238.65)	32.70(9.18,88.73)	**<0.001**
PRL (mIU/L)	282.55(211.50,395.50)	236.00(169.00,335.35)	211.90(150.70,302.00)	**<0.001**
TSTO (nmoL/L)	0.55(0.33,0.84)	0.59(0.33,0.91)	0.52(0.30,0.87)	0.6
LH (IU/L)	7.00(5.24,10.14)	17.11(11.86,26.02)	43.32(33.20,55.68)	**<0.001**
FSH (IU/L)	13.8(11.5,18.0)	31.9(27.4,36.0)	74.2(54.4,96.8)	**<0.001**
PRG (nmoL/L)	0.75(0.44,1.22)	0.71(0.44,1.36)	0.56(0.33,0.89)	**0.036**

The bolded text indicates P<0.05.

**Table 2:** Independent predictors of the occurrence of premature ovarian insufficiency (POI) in the reproductive-aged women analyzed by binary logistic regression.

Characteristics	B	Wald X2	Exp(B)	Between-group P
Age	-1.908	4.372	0.148	0.037
AMH (ng/mL)	-5.490	205.492	0.004	**<0.001**
E2(pmoL/L)	-0.027	42.440	0.973	**<0.001**
Age by ln_ Age	0.415	4.147	1.514	0.042
AMH by ln_ AMH	2.810	173.693	16.608	**<0.001**
E2 by ln_E2	0.004	34.335	1.004	**<0.001**

The bolded text indicates P<0.05.

**Table 3:** Independent predictors of the occurrence of premature ovarian failure (POF) in the reproductive-aged women analyzed by binary logistic regression.

Characteristics	B	Wald X2	Exp(B)	Between-group P
Age	-0.503	0.379	0.605	0.538
AMH (ng/mL)	-0.035	67.026	0.965	**<0.001**
E2 (pmoL/L)	-5.973	127.139	0.003	**<0.001**
Age by ln_ Age	0.099	0.293	1.104	0.588
AMH by ln_ AMH	2.742	110.663	15.516	**<0.001**
E2 by ln_E2	0.005	53.703	1.005	**<0.001**

The bolded text indicates P<0.05.

**Table 4:** AMH, Age, E2, combination of Age and AMH, combination of Age and E2, combination of AMH and E2, combination of Age, AMH and E2 as ROC curves for the development of POI in the reproductive-aged women.

Characteristics	AUC value	95%CI	Cut-off value	Sensitivity (%)	Specificity (%)
Age	0.494	0.460-0.528	39.5	84.3	11.8
AMH	0.885	0.864-0.906	0.040	81.8	85.3
E2	0.757	0.729-0.786	73.00	62.7	83.7
Age+ AMH	0.888	0.868-0.909	–	81.3	85.1
Age+ E2	0.756	0.728-0.785	–	62.7	83.7
AMH+E2	0.909	0.892-0.927	–	82.1	87.5
Age+AMH+E2	0.910	0.893-0.927	–	83.1	86.3

**Table 5:** AMH, Age, E2, combination of Age and AMH, combination of Age and E2, combination of AMH and E2, combination of Age, AMH and E2 as ROC curves for the development of POF in the reproductive-aged women.

Characteristics	AUC value	95%CI	Cut-off value	Sensitivity (%)	Specificity (%)
Age	0.526	0.492-0.561	29.5	19.1	86.5
AMH	0.872	0.849-0.894	0.024	83.5	81.6
E2	0.803	0.775-0.831	73.00	72.0	80.0
Age+ AMH	0.877	0.856-0.898	–	84.2	82.3
Age+ E2	0.805	0.777-0.832	–	68.8	83.1
AMH+E2	0.910	0.892-0.928	–	88.1	80.9
Age+AMH+E2	0.912	0.895-0.930	–	85.0	85.0

## 4 Discussion

AMH is a glycoprotein secreted by granulosa cells of ovarian preantral and small antral follicles, as well as by immature Sertoli cells in the testes (28, 29). In females, AMH plays a critical regulatory role by inhibiting the recruitment of primordial follicles and the development of antral follicles, thereby preventing premature depletion of the ovarian follicle pool (30–32). Compared to traditional biological markers, AMH demonstrates significant advantages in assessing ovarian reserve. It provides earlier indication of age-related decline in ovarian function and exhibits remarkable stability, being largely unaffected by menstrual cycle phase, hormonal contraceptive use, or pregnancy (33). This characteristic has led to its increasing application in gynecological diagnostics. Recent studies highlight AMH’s strong predictive capacity for menopause and premature ovarian insufficiency (34, 35). Notably, serum AMH levels decline prior to the onset of menstrual irregularities or elevations in FSH levels (11). While low AMH concentrations correlate with increased risk of ovarian aging and menopausal transition, its utility as a standalone diagnostic marker for menopause requires further validation (36). In the realm of reproductive medicine, AMH shows particular promise in the diagnosis (37) and pathophysiology (38) of adolescent PCOS. Furthermore, emerging evidence suggests AMH’s involvement in various clinical contexts, including oncological applications (39, 40) (e.g., ovarian cancer biomarker), prevention of age-related bone loss in women (41), management of gynecological endocrine disorders (40), and optimization of assisted reproductive technology (ART) outcomes (33). This multifaceted hormone continues to attract research attention due to its diagnostic potential across reproductive aging, fertility preservation, and endocrine health domains. Continued investigation is warranted to fully elucidate its clinical applications and physiological mechanisms.

Despite garnering significant attention for its diagnostic potential across multiple domains in obstetrics and gynecology, AMH remains underutilized as a validated diagnostic indicator in most clinical practices and international guidelines. For example, while the latest WHO infertility management guidelines explicitly recognize AMH’s pivotal role in assessing female ovarian reserve and fertility potential, they stop short of endorsing it as a primary diagnostic criterion (42). This paradox stems from several unresolved challenges. First, although AMH effectively reflects ovarian function, ovarian aging is intrinsically age-dependent, necessitating precise age-stratified AMH threshold determination to enhance diagnostic accuracy. Second, ovarian function is susceptible to modulation by systemic diseases (e.g., autoimmune disorders, thyroid dysfunction), raising critical questions about how to optimize AMH interpretation in patients with comorbid conditions when diagnosing POI or POF. Third, population-specific variations in AMH levels—including ethnic, genetic, and environmental influences—remain incompletely characterized, limiting the generalizability of existing reference ranges. These interrelated factors collectively impede the translational integration of AMH into routine clinical practice, highlighting the urgent need for large-scale, longitudinal studies to establish standardized, age-adjusted reference intervals and validate its diagnostic performance across diverse patient populations and comorbid conditions. Such efforts are essential to bridge the gap between its biological promise and clinical applicability, ultimately enabling more precise, personalized reproductive healthcare.

AMH, a relatively novel biomarker for ovarian function assessment, remains underutilized in current clinical and diagnostic practices. This study demonstrates that AMH exhibits robust predictive performance in the diagnosis of both POI and POF, with area under the curve (AUC) values exceeding 0.8 in receiver operating characteristic (ROC) analyses (see **Figures 1**, **2**). These findings highlight AMH’s high diagnostic accuracy for POI/POF, which facilitates early disease detection, improves diagnostic rates, enhances reproductive outcomes, elevates quality of life, and reduces long-term health complications and associated burdens. The diagnostic significance observed in this study aligns with previous research findings (21); however, notable discrepancies exist in the diagnostic thresholds. These variations may stem from factors such as regional differences (Southern versus Northern China), assay methodologies, and population heterogeneity. Such discrepancies also guide our future research directions, which should prioritize multi-factorial, stratified investigations across different regions, age groups, and ethnicities.

Compared to traditional diagnostic approaches based on FSH levels and requiring testing during days 2–4 of the menstrual cycle, AMH offers significantly greater convenience in clinical assessment, as it enables measurement at any point throughout a woman’s menstrual cycle. Additionally, prior research has shown that AMH-based diagnostic strategies for PCOS can re-duce total diagnostic costs while optimizing management of secondary comorbidities (43). It is therefore reasonable to hypothesize that AMH may also similarly lower diagnostic expenses and alleviate disease burden in POI/POF contexts. Although age demonstrated a non-significant effect in the diagnosis of POI/POF in this study, the combined diagnostic approach integrating age with AMH and E2 showed superior diagnostic efficacy compared to using AMH or E2 alone. This finding underscores the importance of considering age as a relevant factor in the diagnostic evaluation of POI and POF. Existing literature indicates that AMH levels exhibit age-dependent variations: peaking around 15.8 years of age, maintaining relative stability thereafter, and demonstrating a negative correlation with age beyond 25 years (44, 45). However, our study did not capture this age-related trend, likely due to the narrow age range (30–40 years) of our cohort, which lacked adequate stratification across different age groups to reveal such patterns. As a retrospective chart review, this study was inherently limited in the scope of variables collected. Key clinical parameters such as menstrual cycle regularity, body mass index (BMI), smoking history, and other lifestyle factors—known to influence AMH levels—were not systematically evaluated (46, 47). These limitations highlight critical directions for future research, including prospective cohort designs with broader age distributions, comprehensive clinical profiling, and multivariate analyses to better elucidate the interplay between age, hormonal biomarkers, and ovarian function in the context of POI/POF pathogenesis and diagnosis.

## 5 Limitation

This study is a large-sample, single-center cross-sectional investigation. However, several limitations merit consideration. Due to disease progression and limitations in AMH detection sensitivity, a significant proportion of patients in this study exhibited AMH levels <0.01 U/L, leading to considerable data skew during analytical processes. Additionally, the study population was restricted to individuals with follicle-stimulating hormone (FSH) >10 U/L, which precluded comprehensive investigation across all relevant populations (both affected and healthy individuals). Consequently, the diagnostic accuracy of our findings may be somewhat compromised. Additionally, the limited scope of factors analyzed in this study—such as lifestyle variables, age stratification, and regional disparities—has resulted in an incomplete investigation. This methodological constraint restricts the generalizability of our findings and underscores the need for more comprehensive exploratory frameworks in future research. To address this gap, subsequent investigations will systematically evaluate the diagnostic contributions of these understudied variables, including lifestyle parameters (e.g., smoking status, physical activity, dietary patterns), age-specific biomarker dynamics, and geographic population differences. Such multi-dimensional analyses will enable the development of more precise diagnostic algorithms that account for the heterogeneous etiology of POI and POF, ultimately enhancing clinical decision-making through personalized risk stratification and tailored intervention strategies.

## 6 Conclusion

As an exploratory investigation, this study identified that AMH demonstrated notable diagnostic superiority when employed as a single biomarker for POI and POF. This finding aligns with existing evidence supporting AMH as a robust marker of ovarian reserve due to its relative stability across the menstrual cycle and age-specific expression patterns. Furthermore, our analysis revealed that multi-marker diagnostic approaches—integrating AMH with other hormonal (e.g., E2) and clinical parameters—significantly outperformed single-marker strategies. This synergistic effect underscores the multifactorial nature of ovarian insufficiency, where diminished function arises from the complex interplay of genetic, hormonal, metabolic, and environmental factors rather than isolated biomarker deviations. The implications of these findings extend beyond diagnostic optimization. They emphasize the necessity of adopting holistic diagnostic frameworks that account for the heterogeneity of POI/POF pathogenesis. Future research should prioritize prospective cohort studies incorporating comprehensive clinical profiling (including menstrual history, BMI, lifestyle factors, and genetic markers) alongside advanced statistical modeling to delineate the relative contributions of each factor and their interactions.

In conclusion, while AMH remains a cornerstone biomarker for ovarian function assessment, the superior performance of multi-marker models highlights the need for paradigm shifts in clinical diagnostics—from reductionist single-marker approaches to integrative, patient-centered evaluations. This evolution in diagnostic strategy holds promise for improving diagnostic accuracy, personalizing therapeutic interventions, and ultimately enhancing reproductive outcomes for women affected by ovarian insufficiency.

## Data Availability

The raw data supporting the conclusions of this article will be made available by the authors, without undue reservation.

## References

[1] Touraine P Chabbert-Buffet N Plu-Bureau G Duranteau L Sinclair AH Tucker EJ . Premature ovarian insufficiency. Nat Rev Dis Primers. (2024) 10:63. doi: 10.1038/s41572-024-00547-5, PMID: 39266563

[2] Nash Z Davies M . Premature ovarian insufficiency. BMJ. (2024) 384:e077469. doi: 10.1136/bmj-2023-077469, PMID: 38508679

[3] European Society for Human Reproduction and Embryology (ESHRE) Guideline Group on POI Webber L Davies M Anderson R Bartlett J . ESHRE guideline: management of women with premature ovarian insufficiency. Hum Reprod. (2016) 31:926–37. doi: 10.1093/humrep/dew027, PMID: 27008889

[4] Pastore LM Christianson MS Stelling J Kearns WG Segars JH . Reproductive ovarian testing and the alphabet soup of diagnoses: DOR, POI, POF, POR, and FOR. J Assist Reprod Genet. (2018) 35:17–23. doi: 10.1007/s10815-017-1058-4, PMID: 28971280 PMC5758472

[5] Wang X Wang L Xiang W . Mechanisms of ovarian aging in women: A review. J Ovarian Res. (2023) 16:67. doi: 10.1186/s13048-023-01151-z, PMID: 37024976 PMC10080932

[6] Park SU Walsh L Berkowitz KM . Mechanisms of ovarian aging. Reproduction. (2021) 162:R19–33. doi: 10.1530/REP-21-0022, PMID: 33999842 PMC9354567

[7] Guo T Liu H Xu B Qi Y Xu K Wu X . Epidemiology, genetic etiology and intervention of premature ovarian insufficiency. Endocrine Rev. (2025) 46:621–51. doi: 10.1210/endrev/bnaf011 40177739

[8] Federici S Rossetti R Moleri S Munari EV Frixou M Bonomi M . Primary ovarian insufficiency: update on clinical and genetic findings. Front Endocrinol. (2024) 15:1464803. doi: 10.3389/fendo.2024.1464803, PMID: 39391877 PMC11466302

[9] Li M Zhu Y Wei J Chen L Chen S Lai D . The global prevalence of premature ovarian insufficiency: A systematic review and meta-analysis. Climacteric. (2023) 26:95–102. doi: 10.1080/13697137.2022.2153033, PMID: 36519275

[10] Chon SJ Umair Z Yoon MS . Premature ovarian insufficiency: past, present, and future. Front Cell Dev Biol. (2021) 9:672890. doi: 10.3389/fcell.2021.672890, PMID: 34041247 PMC8141617

[11] Rahman R Panay N . Diagnosis and management of premature ovarian insufficiency. Best Pract Res Clin Endocrinol Metab. (2021) 35:101600. doi: 10.1016/j.beem.2021.101600, PMID: 34823999

[12] Armeni E Paschou SA Goulis DG Lambrinoudaki I . Hormone therapy regimens for managing the menopause and premature ovarian insufficiency. Best Pract Res Clin Endocrinol Metab. (2021) 35:101561. doi: 10.1016/j.beem.2021.101561, PMID: 34274232

[13] Mishra GD Davies MC Hillman S Chung HF Roy S Maclaran K . Optimising health after early menopause. Lancet. (2024) 403:958–68. doi: 10.1016/s0140-6736(23)02800-3, PMID: 38458215

[14] França MM Mendonca BB . Genetics of ovarian insufficiency and defects of folliculogenesis. Best Pract Res Clin Endocrinol Metab. (2022) 36:101594. doi: 10.1016/j.beem.2021.101594, PMID: 34794894

[15] Sullivan SD Sarrel PM Nelson LM . Hormone replacement therapy in young women with primary ovarian insufficiency and early menopause. Fertil Steril. (2016) 106:1588–99. doi: 10.1016/j.fertnstert.2016.09.046, PMID: 27912889 PMC5137796

[16] Dimmick H Kumar TR . Follicle-stimulating hormone. Trends Endocrinol Metab. (2024) 35:848–9. doi: 10.1016/j.tem.2024.04.020, PMID: 39255754 PMC11387984

[17] Palermo R . Differential actions of FSH and LH during folliculogenesis. Reprod BioMed Online. (2007) 15:326–37. doi: 10.1016/s1472-6483(10)60347-1, PMID: 17854533

[18] Recchia K Jorge AS Pessôa LV de F Botigelli RC Zugaib VC de Souza AF . Actions and roles of FSH in germinative cells. Int J Mol Sci. (2021) 22:10110. doi: 10.3390/ijms221810110, PMID: 34576272 PMC8470522

[19] Oduwole OO Huhtaniemi IT Misrahi M . The roles of luteinizing hormone, follicle-stimulating hormone and testosterone in spermatogenesis and folliculogenesis revisited. IJMS. (2021) 22:12735. doi: 10.3390/ijms222312735, PMID: 34884539 PMC8658012

[20] ESHRE ASRM CREWHIRL and IMS Guideline Group on POI Panay N Anderson RA Bennie A . Evidence-based guideline: premature ovarian insufficiency. Fertil Steril. (2025) 123:221–36. doi: 10.1016/j.fertnstert.2024.11.007, PMID: 39652037

[21] Jiao X Meng T Zhai Y Zhao L Luo W Liu P . Ovarian reserve markers in premature ovarian insufficiency: within different clinical stages and different etiologies. Front Endocrinol. (2021) 12:601752. doi: 10.3389/fendo.2021.601752, PMID: 33815272 PMC8015703

[22] Tal R Seifer DB . Ovarian reserve testing: A user’s guide. Am J Obstet Gynecol. (2017) 217:129–40. doi: 10.1016/j.ajog.2017.02.027, PMID: 28235465

[23] Di Clemente N Racine C Pierre A Taieb J . Anti-müllerian hormone in female reproduction. Endocrine Rev. (2021) 42:753–82. doi: 10.1210/endrev/bnab012 33851994

[24] Erel CT Ozcivit IB . Anti-müllerian hormone and ovarian aging. Gynecol Endocrinol. (2021) 37:867–8. doi: 10.1080/09513590.2021.1977276, PMID: 34519604

[25] Moolhuijsen LME Visser JA . Anti-müllerian hormone and ovarian reserve: update on assessing ovarian function. J Clin Endocrinol Metab. (2020) 105:3361–73. doi: 10.1210/clinem/dgaa513, PMID: 32770239 PMC7486884

[26] Huang Y Kuang X Jiangzhou H Li M Yang D Lai D . Using anti-müllerian hormone to predict premature ovarian insufficiency: A retrospective cross-sectional study. Front Endocrinol. (2024) 15:1454802. doi: 10.3389/fendo.2024.1454802, PMID: 39629049 PMC11611575

[27] Expert Group of Consensus on Clinical Diagnosis & Management of Diminished Ovarian Reserve & Reproductive Endocrinology & Fertility Preservation Section of Chinese Society on Fertility Preservation under Chinese Preventive Medicine Association . Consensus on clinical diagnosis and management of diminished ovarian reserve. J Reprod Med. (2022) 31:425–34. doi: 10.3969/j.issn.1004-3845.2022.04.001

[28] La Marca A Broekmans FJ Volpe A Fauser BC Macklon NS on behalf of the ESHRE Special Interest Group for Reproductive Endocrinology - AMH Round Table . Anti-mullerian hormone (AMH): what do we still need to know? Hum Reprod. (2009) 24:2264–75. doi: 10.1093/humrep/dep210, PMID: 19520713

[29] Muñoz-Jurado A Requena F Agüera EI Escribano BM . Role of anti-müllerian hormone in different reproductive aspects of female mammals: women, cow and mare. Anim Health Res Rev. (2023) 24:64–74. doi: 10.1017/S1466252324000021, PMID: 40123547

[30] Broekmans FJ Visser JA Laven JSE Broer SL Themmen APN Fauser BC . Anti-müllerian hormone and ovarian dysfunction. Trends Endocrinol Metab. (2008) 19:340–7. doi: 10.1016/j.tem.2008.08.002, PMID: 18805020

[31] Buratini J Dellaqua TT Dal Canto M La Marca A Carone D Mignini Renzini M . The putative roles of FSH and AMH in the regulation of oocyte developmental competence: from fertility prognosis to mechanisms underlying age-related subfertility. Hum Reprod Update. (2022) 28:232–54. doi: 10.1093/humupd/dmab044, PMID: 34969065

[32] Iwase A Hasegawa Y Tsukui Y Kobayashi M Hiraishi H Nakazato T . Anti-müllerian hormone beyond an ovarian reserve marker: the relationship with the physiology and pathology in the life-long follicle development. Front Endocrinol. (2023) 14:1273966. doi: 10.3389/fendo.2023.1273966, PMID: 38027144 PMC10657644

[33] Russell N Gilmore A Roudebush WE . Clinical utilities of anti-müllerian hormone. JCM. (2022) 11:7209. doi: 10.3390/jcm11237209, PMID: 36498783 PMC9741321

[34] Laven JSE Louwers YV . Can we predict menopause and premature ovarian insufficiency? Fertil Steril. (2024) 121:737–41. doi: 10.1016/j.fertnstert.2024.02.029, PMID: 38382699

[35] Long P Tan H Chen B Wang L Quan R Hu Z . Dissecting the shared genetic architecture between anti-müllerian hormone and age at menopause based on genome-wide association study. Am J Obstet Gynecol. (2024) 231:634.e1–634.e11. doi: 10.1016/j.ajog.2024.06.050, PMID: 38969199 PMC12038692

[36] Nelson SM Davis SR Kalantaridou S Lumsden MA Panay N Anderson RA . Anti-müllerian hormone for the diagnosis and prediction of menopause: A systematic review. Hum Reprod Update. (2023) 29:327–46. doi: 10.1093/humupd/dmac045, PMID: 36651193 PMC10152172

[37] Tsukui Y Kitahara Y Hasegawa Y Kobayashi M Osuka S Iwase A . Anti-müllerian hormone levels in the diagnosis of adolescent polycystic ovarian syndrome: A systematic review and meta-analysis. Endocr J. (2022) 69:897–906. doi: 10.1507/endocrj.EJ22-0081, PMID: 35675999

[38] Dewailly D Barbotin A-L Dumont A Catteau-Jonard S Robin G . Role of anti-müllerian hormone in the pathogenesis of polycystic ovary syndrome. Front Endocrinol. (2020) 11:641. doi: 10.3389/fendo.2020.00641, PMID: 33013710 PMC7509053

[39] Anderson RA Cameron D Clatot F Demeestere I Lambertini M Nelson SM . Anti-müllerian hormone as a marker of ovarian reserve and premature ovarian insufficiency in children and women with cancer: A systematic review. Hum Reprod Update. (2022) 28:417–34. doi: 10.1093/humupd/dmac004, PMID: 35199161 PMC9071067

[40] Gowkielewicz M Lipka A Zdanowski W Waśniewski T Majewska M Carlberg C . Anti-müllerian hormone: biology and role in endocrinology and cancers. Front Endocrinol. (2024) 15:1468364. doi: 10.3389/fendo.2024.1468364, PMID: 39351532 PMC11439669

[41] Karlamangla AS Shieh A Greendale GA Yu EW Burnett-Bowie SAM Sluss PM . Anti-mullerian hormone as predictor of future and ongoing bone loss during the menopause transition. J Bone Mineral Res. (2020) 37:1224–32. doi: 10.1002/jbmr.4525, PMID: 35373854 PMC9283201

[42] World Health Organization . Guideline for the prevention, diagnosis and treatment of infertility. Geneva: World Health Organization (2025). Available online at: https://www.who.int/publications/i/item/9789240115774, ISBN: ISBN 978-92-4-011577-4.

[43] Garay OU Olziersky AM Laven J Mawson R Piltonen T Franks S . Economic impact of the elecsys an-ti-müllerian hormone plus immunoassay for anti-müllerian hormone testing as part of polycystic ovary syndrome assess-ment in the United Kingdom. PloS One. (2025) 20:e0326162. doi: 10.1371/journal.pone.0326162, PMID: 40526694 PMC12173178

[44] Lie FS Visser JA Welt CK de Rijke YB Eijkemans MJ Broekmans FJ . Serum anti-müllerian hormone levels in healthy females: a nomogram ranging from infancy to adulthood. J Clin Endocrinol Metab. (2012) 97:4650–5. doi: 10.1210/jc.2012-1440, PMID: 22993032 PMC3683801

[45] Aslan K Kasapoglu I Kosan B Tunali A Tellioglu I Uncu G . Age-stratified anti-Müllerian hormone (AMH) nomogram: a comprehensive cohort study including 22.920 women. Front Endocrinol (Lausanne). (2025) 16:1612194. doi: 10.3389/fendo.2025.1612194, PMID: 40620793 PMC12226283

[46] Werner L van der Schouw YT de Kat AC . A systematic review of the association between modifiable lifestyle factors and circulating anti-Müllerian hormone. Hum Reprod Update. (2024) 30:262–308. doi: 10.1093/humupd/dmae004, PMID: 38402486

[47] Younis JS Iskander R Fauser BCJM Izhaki I . Does an association exist between menstrual cycle length within the normal range and ovarian reserve biomarkers during the reproductive years? A systematic review and meta-analysis. Hum Reprod Update. (2020) 26:904–28. doi: 10.1093/humupd/dmaa013, PMID: 32514566

