# Supplementary material for: Diagnostic accuracy of AMH for primary ovarian insufficiency/premature ovarian failure: a real-world cohort study
Source: Front Endocrinol (Lausanne). 2026 Feb 11;17:1742145. doi: 10.3389/fendo.2026.1742145 (PMC12932242; doi:10.3389/fendo.2026.1742145)
Supplement: Supplementary file 1 [file DataSheet1.zip › Quality Control Certificates/Value Sheet.PreciControl AMH-2016-10.pdf]

AMH

Anti-Müllerian hormone

REF	Σ	SYSTEM
**06331076** 190	100	Elecsys 2010<br>MODULAR ANALYTICS E170<br>**cobas e** 411<br>**cobas e** 601<br>**cobas e** 602

English

System information

For **cobas e** 411 analyzer: test number 1130
For MODULAR ANALYTICS E170, **cobas e** 601 and **cobas e** 602 analyzers: Application Code Number 782

Intended use

Immunoassay for the in vitro quantitative determination of anti-Müllerian hormone (AMH) in human serum and plasma. The determination of AMH is used for the assessment of the ovarian reserve and the prediction of response to controlled ovarian stimulation (COS) in conjunction with other clinical and laboratory findings.

The **e**lectro**c**hemi**l**uminescence **i**mmuno**a**ssay "ECLIA" is intended for use on Elecsys and **cobas e** immunoassay analyzers.

Summary

The anti-Müllerian hormone is a homodimeric glycoprotein belonging to the transforming growth factor β (TGF β) family. All members of this superfamily are involved in the regulation of tissue growth and differentiation. Prior to secretion, the hormone undergoes glycosylation and dimerization to produce an approximately 140 kDa precursor of two identical disulfide-linked 70 kDa subunits. Each monomer contains a large N-terminal pro-region and a much smaller C-terminal mature domain. In contrast to other TGF β family members, AMH is thought to require the N-terminal domain to potentiate activity of the C-terminal domain to attain full bioactivity.[1,2]

A part of AMH is then cleaved at a specific site between the pro-region and the mature region during cytoplasmic transit to generate biologically active 110 kDa N-terminal and 25 kDa C-terminal homodimers which remain associated in a non-covalent complex. The AMH type II receptor (AMH RII) has the capacity of binding only the biologically active form of AMH.[2]

In males, AMH is secreted by the Sertoli cells of the testes. During embryonic development in males, secretion of AMH from testicular Sertoli cells is responsible for the regression of the Müllerian duct and the normal development of the male reproductive tract. The secretion of AMH by the Sertoli cells starts during the embryogenesis and continues throughout life. AMH is continuously produced by the testicles until puberty and then decreases slowly to post-puberty values.[3]

In females AMH plays an important role in the ovarian folliculogenesis.[4] Follicle development in the ovaries comprises two distinct stages: initial recruitment, by which primordial follicles start to mature, and cyclic recruitment, which leads to the growth of a cohort of small antral follicles, among which the dominant follicle (destined to ovulate) is subsequently selected. FSH directs the cyclic recruitment. AMH expression in granulosa cells starts in primary follicles and is maximal in granulosa cells of preantral and small antral follicles up to approximately 6 mm in diameter. When follicle growth becomes FSH-dependent, AMH expression diminishes and becomes undetectable. This pattern of AMH expression supports the inhibitory role of AMH at two distinct stages of folliculogenesis. First, AMH inhibits the transition of follicles from primordial into maturation stages and thereby has an important role in regulating the number of follicles remaining in the primordial pool. Second, AMH has inhibitory effects on follicular sensitivity to FSH and therefore has a role in the process of follicular selection.[5,6]

Serum levels of AMH are barely detectable at birth in females, reach their highest levels after puberty, decrease progressively thereafter with age, and become undetectable at menopause.[7,8] Serum AMH levels have been shown to be relatively stable during the menstrual cycle with substantial fluctuations being observed in younger women.[9,10,11] AMH levels further demonstrate lower intra- and inter-cyclic variation than baseline FSH.[10] Serum AMH levels decrease significantly during the use of combined contraceptives.[12] Clinical applications of AMH measurements have been proposed for a variety of indications.[13,14,15] Measurement of serum AMH is clinically mainly used for assessment of ovarian reserve reflecting the number of antral and pre-antral follicles, the so-called antral follicle count (AFC), and for the prediction of response to controlled ovarian stimulation.[13,15,16] Further clinical applications of AMH are diagnosis of disorders of sex development (DSD) in children[17,18] and monitoring of granulosa cell tumors to detect residual or recurrent disease.[19,20] AMH has been suggested as a surrogate biomarker for AFC in the diagnosis of polycystic ovary syndrome (PCOS)[21,22] and for the prediction of time to menopause.[23,24]

Test principle

Sandwich principle. Total duration of assay: 18 minutes.

- 1st incubation: 50 µL of sample, a biotinylated monoclonal AMH-specific antibody, and a monoclonal AMH-specific antibody labeled with a ruthenium complex[a)] form a sandwich complex.
- 2nd incubation: After addition of streptavidin-coated microparticles, the complex becomes bound to the solid phase via interaction of biotin and streptavidin.
- The reaction mixture is aspirated into the measuring cell where the microparticles are magnetically captured onto the surface of the electrode. Unbound substances are then removed with ProCell/ProCell M. Application of a voltage to the electrode then induces chemiluminescent emission which is measured by a photomultiplier.
- Results are determined via a calibration curve which is instrument-specifically generated by 2-point calibration and a master curve provided via the reagent barcode or e-barcode.

a) Tris(2,2'-bipyridyl)ruthenium(II)-complex ($Ru(bpy)_3^{2+}$)

Reagents - working solutions

The reagent rackpack is labeled as AMH.

M Streptavidin-coated microparticles (transparent cap), 1 bottle, 6.5 mL:
Streptavidin-coated microparticles 0.72 mg/mL; preservative.

R1 Anti-AMH-Ab~biotin (gray cap), 1 bottle, 8 mL:
Biotinylated monoclonal anti-AMH antibody (mouse) 1.0 mg/L, phosphate buffer 50 mmol/L, pH 7.5; preservative.

R2 Anti-AMH-Ab~$Ru(bpy)_3^{2+}$ (black cap), 1 bottle, 8 mL:
Monoclonal anti-AMH antibody (mouse) labeled with ruthenium complex 1.0 mg/L, phosphate buffer 50 mmol/L, pH 7.5; preservative.

Precautions and warnings

For in vitro diagnostic use.
Exercise the normal precautions required for handling all laboratory reagents.
Disposal of all waste material should be in accordance with local guidelines.
Safety data sheet available for professional user on request.

Avoid foam formation in all reagents and sample types (specimens, calibrators and controls).

Reagent handling

The reagents in the kit have been assembled into a ready-for-use unit that cannot be separated.

All information required for correct operation is read in from the respective reagent barcodes.

Storage and stability

Store at 2-8 °C.

Do not freeze.

Store the Elecsys reagent kit **upright** in order to ensure complete availability of the microparticles during automatic mixing prior to use.

Stability:	
unopened at 2-8 °C	up to the stated expiration date
after opening at 2-8 °C	12 weeks
on the analyzers	8 weeks

Specimen collection and preparation

Only the specimens listed below were tested and found acceptable.

Serum collected using standard sampling tubes or tubes containing separating gel.

Li-heparin plasma. Do not use EDTA plasma.

Criterion: Recovery within ± 30 % of serum value ≥ 3.57 pmol/L (≥ 0.5 ng/mL); recovery within ± 1.43 pmol/L (± 0.2 ng/mL) for serum value < 3.57 pmol/L (< 0.5 ng/mL) and slope of 0.9-1.1 + intercept within ± 0.7 pmol/L (± 0.1 ng/mL) + coefficient of correlation ≥ 0.95.

Stable for 3 days at 20-25 °C, 5 days at 2-8 °C, 6 months at -20 °C (± 5 °C). Freeze only once.

The sample types listed were tested with a selection of sample collection tubes that were commercially available at the time of testing, i.e. not all available tubes of all manufacturers were tested. Sample collection systems from various manufacturers may contain differing materials which could affect the test results in some cases. When processing samples in primary tubes (sample collection systems), follow the instructions of the tube manufacturer.

Centrifuge samples containing precipitates before performing the assay.

Do not use heat-inactivated samples.

Do not use samples and controls stabilized with azide.

Ensure the samples, calibrators and controls are at 20-25 °C prior to measurement.

Due to possible evaporation effects, samples, calibrators and controls on the analyzers should be analyzed/measured within 2 hours.

Materials provided

See "Reagents – working solutions" section for reagents.

Materials required (but not provided)

- REF 06331084190, AMH CalSet, for 4 x 1 mL
- REF 06709966190, PreciControl AMH, for 2 x 2 mL each of PreciControl AMH 1 and 2
- REF 05192943190, Diluent Universal 2, 2 x 36 mL sample diluent or REF 11732277122, Diluent Universal, 2 x 16 mL sample diluent or REF 03183971122, Diluent Universal, 2 x 36 mL sample diluent
- General laboratory equipment
- Elecsys 2010, MODULAR ANALYTICS E170 or **cobas e** analyzer

Accessories for Elecsys 2010 and **cobas e** 411 analyzers:

- REF 11662988122, ProCell, 6 x 380 mL system buffer
- REF 11662970122, CleanCell, 6 x 380 mL measuring cell cleaning solution
- REF 11930346122, Elecsys SysWash, 1 x 500 mL washwater additive
- REF 11933159001, Adapter for SysClean
- REF 11706802001, AssayCup, 60 x 60 reaction cups
- REF 11706799001, AssayTip, 30 x 120 pipette tips
- REF 11800507001, Clean-Liner

Accessories for MODULAR ANALYTICS E170, **cobas e** 601 and **cobas e** 602 analyzers:

- REF 04880340190, ProCell M, 2 x 2 L system buffer
- REF 04880293190, CleanCell M, 2 x 2 L measuring cell cleaning solution
- REF 03023141001, PC/CC-Cups, 12 cups to prewarm ProCell M and CleanCell M before use
- REF 03005712190, ProbeWash M, 12 x 70 mL cleaning solution for run finalization and rinsing during reagent change
- REF 03004899190, PreClean M, 5 x 600 mL detection cleaning solution
- REF 12102137001, AssayTip/AssayCup, 48 magazines x 84 reaction cups or pipette tips, waste bags
- REF 03023150001, WasteLiner, waste bags
- REF 03027651001, SysClean Adapter M

Accessories for all analyzers:

- REF 11298500316, ISE Cleaning Solution/Elecsys SysClean, 5 x 100 mL system cleaning solution

Assay

For optimum performance of the assay follow the directions given in this document for the analyzer concerned. Refer to the appropriate operator's manual for analyzer-specific assay instructions.

Resuspension of the microparticles takes place automatically prior to use. Read in the test-specific parameters via the reagent barcode. If in exceptional cases the barcode cannot be read, enter the 15-digit sequence of numbers (except for the **cobas e** 602 analyzer).

MODULAR ANALYTICS E170, **cobas e** 601 and **cobas e** 602 analyzers: PreClean M solution is necessary.

Bring the cooled reagents to approximately 20 °C and place on the reagent disk (20 °C) of the analyzer. Avoid foam formation. The system automatically regulates the temperature of the reagents and the opening/closing of the bottles.

Calibration

Traceability: This method has been standardized against the Beckman Coulter AMH Gen II ELISA (unmodified version without predilution) assay.

Every Elecsys reagent set has a barcoded label containing specific information for calibration of the particular reagent lot. The predefined master curve is adapted to the analyzer using the relevant CalSet.

Calibration frequency: Calibration must be performed once per reagent lot using fresh reagent (i.e. not more than 24 hours since the reagent kit was registered on the analyzer). Renewed calibration is recommended as follows:

- after 1 month (28 days) when using the same reagent lot
- after 7 days (when using the same reagent kit on the analyzer)
- as required: e.g. quality control findings outside the defined limits

Quality control

For quality control, use PreciControl AMH.

In addition, other suitable control material can be used.

Controls for the various concentration ranges should be run individually at least once every 24 hours when the test is in use, once per reagent kit, and following each calibration.

The control intervals and limits should be adapted to each laboratory's individual requirements. Values obtained should fall within the defined limits. Each laboratory should establish corrective measures to be taken if values fall outside the defined limits.

Follow the applicable government regulations and local guidelines for quality control.

Calculation

The analyzer automatically calculates the analyte concentration of each sample (either in pmol/L or in ng/mL).

Conversion factors:	pmol/L x 0.14 = ng/mL
	ng/mL x 7.14 = pmol/L

Limitations - interference

The assay is unaffected by icterus (bilirubin ≤ 1129 µmol/L or ≤ 66 mg/dL), hemolysis (Hb ≤ 0.621 mmol/L or ≤ 1.0 g/dL), lipemia (Intralipid ≤ 1000 mg/dL), biotin (≤ 143 nmol/L or ≤ 30 ng/mL), IgG ≤ 2.5 g/dL, IgA ≤ 1.8 g/dL and IgM ≤ 0.5 g/dL.

Criterion: Recovery within ± 10 % of initial value.

Samples should not be taken from patients receiving therapy with high biotin doses (i.e. > 5 mg/day) until at least 8 hours following the last biotin administration.

No interference was observed from rheumatoid factors up to a concentration of 1000 IU/mL.

There is no high-dose hook effect at AMH concentrations up to 9996 pmol/L (1400 ng/mL).

In vitro tests were performed on 20 commonly used pharmaceuticals. No interference with the assay was found up to the concentrations indicated within the below table.

Active Agent	Concentration tested mg/L
Acetylcysteine	150
Ampicillin-Na	1000
Ascorbic acid	300
Cyclosporine	5
Cefoxitin	2500
Heparin	5000 U
Levodopa	20
Methyldopa	20
Metronidazole	200
Phenylbutazone	400
Doxycycline	50
Acetylsalicylic Acid	1000
Rifampicin	60
Acetaminophen	200
Ibuprofen	500
Theophylline	100
Triporelin acetate	0.1
Metformin	2000
Folic Acid	0.4
Levothyroxine	0.2

In rare cases, interference due to extremely high titers of antibodies to analyte-specific antibodies, streptavidin or ruthenium can occur. These effects are minimized by suitable test design.

For diagnostic purposes, the results should always be assessed in conjunction with the patient's medical history, clinical examination and other findings.

Limits and ranges

Measuring range

0.07-164 pmol/L (0.01-23 ng/mL) (defined by the Limit of Detection and the maximum of the master curve). Values below the Limit of Detection are reported as < 0.07 pmol/L (< 0.01 ng/mL). Values above the measuring range are reported as > 164 pmol/L (> 23 ng/mL) or up to 328 pmol/L (46 ng/mL) for 2-fold diluted samples.

Lower limits of measurement

Limit of Blank, Limit of Detection and Limit of Quantitation

Limit of Blank = 0.049 pmol/L (0.007 ng/mL)

Limit of Detection = 0.07 pmol/L (0.01 ng/mL)

Limit of Quantitation = 0.21 pmol/L (0.030 ng/mL)

The Limit of Blank, Limit of Detection and Limit of Quantitation were determined in accordance with the CLSI (Clinical and Laboratory Standards Institute) EP17-A2 requirements.

The Limit of Blank is the 95th percentile value from n ≥ 60 measurements of analyte-free samples over several independent series. The Limit of Blank corresponds to the concentration below which analyte-free samples are found with a probability of 95 %.

The Limit of Detection is determined based on the Limit of Blank and the standard deviation of low concentration samples. The Limit of Detection corresponds to the lowest analyte concentration which can be detected (value above the Limit of Blank with a probability of 95 %).

The Limit of Quantitation is the lowest analyte concentration that can be reproducibly measured with an intermediate precision CV of ≤ 20 %.

Dilution

Samples with AMH concentrations above the measuring range can be diluted automatically with Diluent Universal 2. Manual dilution can be performed with Diluent Universal 2 or Diluent Universal. The recommended dilution is 1:2 (either automatically by the analyzers or manually). The concentration of the diluted sample must be > 71.4 pmol/L (> 10 ng/mL).

After manual dilution, multiply the result by the dilution factor.

After dilution by the analyzers, the software automatically takes the dilution into account when calculating the sample concentration.

Expected values

A study in a Caucasian population with the Elecsys AMH assay on samples from apparently healthy adults (148 males, 887 females not taking contraceptives) and 149 women with Polycystic Ovary Syndrome yielded the following results (Roche study No. RD001727):

	N	2.5th perc. ng/mL (95 % CI$^{b)}$)	5th perc. ng/mL (95 % CI)	Median ng/mL (95 % CI)	95th perc. ng/mL (95 % CI)	97.5th perc. ng/mL (95 % CI)
Healthy men						
	148	0.77 (0.17-1.58)	1.43 (0.256-1.97)	4.79 (4.35-5.35)	11.6 (10.3-17.0)	14.5 (10.9-17.6)
Healthy women (years)						
• 20-24	150	1.22 (0.478-1.67)	1.52 (0.758-1.81)	4.00 (3.60-4.44)	9.95 (7.87-13.6)	11.7 (9.11-15.7)
• 25-29	150	0.890 (0.493-1.21)	1.20 (0.797-1.75)	3.31 (3.00-3.89)	9.05 (7.59-10.3)	9.85 (8.91-11.3)
• 30-34	138	0.576 (0.256-0.958)	0.711 (0.256-1.12)	2.81 (2.35-3.47)	7.59 (6.84-9.52)	8.13 (7.27-9.72)
• 35-39	138	0.147 (0.053-0.474)	0.405 (0.053-0.496)	2.00 (1.73-2.36)	6.96 (5.31-9.37)	7.49 (6.49-10.9)
• 40-44	142	0.027 (0.010-0.063)	0.059 (0.017-0.119)	0.882 (0.726-1.13)	4.44 (2.94-5.56)	5.47 (3.92-6.76)
• 45-50	169	0.010 (0.010-0.010)	0.010 (0.010-0.010)	0.194 (0.144-0.269)	1.79 (1.43-2.99)	2.71 (1.79-4.16)
PCOS women*						
	149	1.86 (1.54-2.50)	2.41 (1.67-3.01)	6.81 (6.30-7.42)	17.1 (13.3-20.3)	18.9 (16.0-21.1)

b) CI = confidence interval

	N	2.5th perc. pmol/L (95 % CI)	5th perc. pmol/L (95 % CI)	Median pmol/L (95 % CI)	95th perc. pmol/L (95 % CI)	97.5th perc. pmol/L (95 % CI)
Healthy men						
	148	5.5 (1.2-11.3)	10.2 (1.8-14.1)	34.2 (31.1-38.2)	82.8 (73.5-121)	103 (78.1-125)
Healthy women (years)						
• 20-24	150	8.71 (3.41-11.9)	10.9 (5.41-12.9)	28.6 (25.7-31.7)	71.0 (56.2-97.1)	83.6 (65.0-112)

AMH

Anti-Müllerian hormone

	N	2.5th perc. pmol/L (95 % CI)	5th perc. pmol/L (95 % CI)	Median pmol/L (95 % CI)	95th perc. pmol/L (95 % CI)	97.5th perc. pmol/L (95 % CI)
• 25-29	150	6.35 (3.52-8.64)	8.57 (5.69-12.5)	23.6 (21.4-27.8)	64.6 (54.2-73.5)	70.3 (63.6-81.0)
• 30-34	138	4.11 (1.83-6.84)	5.08 (1.83-8.00)	20.0 (16.8-24.8)	54.2 (48.8-68.0)	58.0 (51.9-69.4)
• 35-39	138	1.05 (0.378-3.38)	2.89 (0.378-3.54)	14.2 (12.4-16.9)	49.7 (37.9-66.9)	53.5 (46.3-77.9)
• 40-44	142	0.193 (0.071-0.450)	0.421 (0.121-0.850)	6.29 (5.18-8.07)	31.7 (21.0-39.7)	39.1 (28.0-48.3)
• 45-50	169	0.071 (0.071-0.071)	0.071 (0.071-0.071)	1.39 (1.03-1.92)	12.8 (10.2-21.3)	19.3 (12.8-29.7)
PCOS women*						
	149	13.3 (11.0-17.8)	17.2 (11.9-21.5)	48.6 (45.0-53.0)	122 (95.0-145)	135 (114-151)

* According to the revised diagnostic criteria of PCOS defined by the Rotterdam ESHRE/ASRM-sponsored (ESHRE = European Society of Human Reproduction and Embryology; ASRM = American Society of Reproductive Medicine) PCOS consensus workshop group.[25]

Each laboratory should investigate the transferability of the expected values to its own patient population and if necessary determine its own reference ranges.

Use of AMH for the assessment of ovarian reserve

The use of AMH for the assessment of ovarian reserve was investigated in a prospective study with n = 451 women between 18-44 years old, where AMH values were correlated to the antral follicle count (AFC) of the women (Roche study No. RD001542). AFC was determined by transvaginal sonography measuring follicles of 2-10 mm diameter in size. Both AFC and AMH were determined on days 2-4 of the same menstrual cycle. Between 17 to 115 women were recruited per site at 6 different European sites and 1 Australian site.

No significant differences in mean AMH values were observed between the sites (pval = 0.301). The mean age values between the sites were significantly different, and also AMH and age showed a significant negative correlation (Spearman correlation coefficient -0.47). The age adjusted site effect of AMH showed no significance (pval = 0.193). The determined AFC values showed significant differences between the sites, with and without age adjustment. The overall correlation of AMH with AFC was 0.68 (Spearman's rank coefficient).

The figure below shows the scatterplot of AMH versus AFC, as well as the site-specific AMH and AFC distributions.

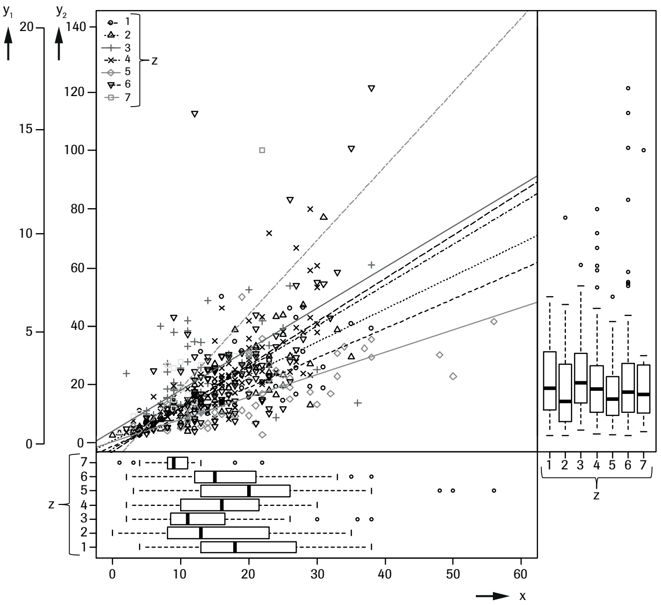

x: AFC (N)

y_1: AMH (ng/mL)

y_2: AMH (pmol/L)

z: Site

Agreement table on absolute AFC numbers of 7 and 15

Three AFC groups were defined[26,27] based on two cutoffs for AFC: 7 and 15 (0-7, 8-15, > 15). According to the prevalences within these groups (15 %, 37 %, 48 %), quantiles on AMH were computed (c1 = 4.86 pmol/L or 0.681 ng/mL, c2 = 16.2 pmol/L or 2.27 ng/mL) to define three groups. Agreement is presented in absolute numbers and percentages per AMH group.

Given the large variability of AFC results depending on site- and sonographer-specific variations, each site should review the agreement table for transferability to their own specific conditions.

	AFC 0-7	AFC 8-15	AFC > 15	N
AMH ≤ 4.86 pmol/L (0.681 ng/mL)	43 (63.2 %)	22 (32.4 %)	3 (4.4 %)	68
4.86 pmol/L (0.681 ng/mL) < AMH ≤ 16.2 pmol/L (2.27 ng/mL)	20 (12.0 %)	95 (56.9 %)	52 (31.1 %)	167
AMH > 16.2 pmol/L (2.27 ng/mL)	3 (1.4 %)	52 (24.1 %)	161 (74.5 %)	216
N	66	169	216	451

For a patient with AMH ≤ 4.86 pmol/L (0.681 ng/mL) the probability to have a low AFC (0-7) is 63 %, the probability to be in the middle AFC (8-15) group is about 32 % and only 4.4 % for having an AFC > 15.

The probability for patients with high AMH values (> 16.2 pmol/L; 2.27 ng/mL) to have an AFC > 15 is 75 %, the probability for being in the middle AFC (8-15) group is 24 % and only 1.4 % to have an AFC < 8.

Use of AMH for the prediction of hyper-response to controlled ovarian stimulation

The following results were obtained in an external study "Clinical evaluation of the Elecsys AMH assay for the prediction of response to controlled ovarian stimulation" (Roche study No. CIM RD 001695).

AMH was determined in 149 women undergoing an antagonist treatment protocol in the course of their first cycle of controlled ovarian stimulation for in-vitro fertilization (IVF). Women included in the study were aged < 44 years, had a regular menstruation cycle and no major abnormalities at transvaginal sonography. Women with PCOS, endocrine or metabolic abnormalities and women undergoing IVF with oocyte donation were not included. All women received a standard FSH stimulation dose of

150 IU/day. Blood was drawn before start of FSH stimulation for post hoc analysis of AMH after completion of the treatment cycle. Hyper-response was observed in 16 women. Hyper-response was defined as > 15 oocytes retrieved or cancellation of stimulation cycle where more than 20 follicles > 12 mm were observed and Estradiol values > 11700 pmol/L, or when more than 30 follicles > 12 mm were observed. The clinical performance of Elecsys AMH to predict hyper-response to controlled ovarian stimulation was evaluated by ROC (receiver operating characteristic) analysis and by applying a cutoff of 15 pmol/L (2.1 ng/mL) which has been previously published.[28,29] Prediction of hyper-response was significant with an AUC (area under the curve) of 82.1 % (CI 72.5-91.7 %). Sensitivity, specificity, positive predictive value (PPV), and negative predictive value (NPV) for the AMH cutoff of 15.0 pmol/L (2.1 ng/mL) are shown in the table below.

**Hyper-response**		
AMH cutoff	15.0 pmol/L (2.10 ng/mL)	
	Estimate	95 % CI
Sensitivity	81.3 %	54.4-96.0 %
Specificity	64.7 %	55.9-72.8 %
PPV	21.7 %	12.1-34.2 %
NPV	96.6 %	90.5-99.3 %

Specific performance data

Representative performance data on the analyzers are given below. Results obtained in individual laboratories may differ.

Precision

Precision was determined using Elecsys reagents, samples and controls in a protocol (EP5-A2) of the CLSI (Clinical and Laboratory Standards Institute): 2 runs per day in duplicate each for 21 days (n = 84). The following results were obtained:

Elecsys 2010 and **cobas e** 411 analyzers					
		**Repeatability**		**Intermediate precision**	
Sample	Mean ng/mL	SD ng/mL	CV %	SD ng/mL	CV %
Human serum 1	0.232	0.004	1.8	0.010	4.4
Human serum 2	0.705	0.008	1.1	0.028	4.0
Human serum 3	2.44	0.030	1.2	0.082	3.3
Human serum 4	12.3	0.132	1.1	0.449	3.7
Human serum 5	18.8	0.287	1.5	0.711	3.8
PreciControl AMH 1	1.15	0.011	1.0	0.033	2.9
PreciControl AMH 2	5.68	0.057	1.0	0.214	3.8

MODULAR ANALYTICS E170, **cobas e** 601 and **cobas e** 602 analyzers					
		**Repeatability**		**Intermediate precision**	
Sample	Mean ng/mL	SD ng/mL	CV %	SD ng/mL	CV %
Human serum 1	0.248	0.004	1.7	0.008	3.2
Human serum 2	0.745	0.009	1.2	0.020	2.7
Human serum 3	2.55	0.028	1.1	0.070	2.7
Human serum 4	13.0	0.165	1.3	0.397	3.0
Human serum 5	19.6	0.188	1.0	0.650	3.3
PreciControl AMH 1	1.19	0.014	1.2	0.042	3.5
PreciControl AMH 2	5.89	0.055	0.9	0.200	3.4

Elecsys 2010 and **cobas e** 411 analyzers					
		**Repeatability**		**Intermediate precision**	
Sample	Mean pmol/L	SD pmol/L	CV %	SD pmol/L	CV %
Human serum 1	1.6	0.030	1.8	0.072	4.4
Human serum 2	5.03	0.054	1.1	0.200	4.0
Human serum 3	17.4	0.213	1.2	0.584	3.3
Human serum 4	87.5	0.941	1.1	3.20	3.7
Human serum 5	134	2.05	1.5	5.08	3.8
PreciControl AMH 1	8.19	0.082	1.0	0.23	2.9
PreciControl AMH 2	40.5	0.405	1.0	1.53	3.8

MODULAR ANALYTICS E170, **cobas e** 601 and **cobas e** 602 analyzers					
		**Repeatability**		**Intermediate precision**	
Sample	Mean pmol/L	SD pmol/L	CV %	SD pmol/L	CV %
Human serum 1	1.7	0.030	1.7	0.057	3.2
Human serum 2	5.32	0.065	1.2	0.142	2.7
Human serum 3	18.2	0.197	1.1	0.497	2.7
Human serum 4	93.1	1.18	1.3	2.84	3.0
Human serum 5	140	1.34	1.0	4.64	3.3
PreciControl AMH 1	8.49	0.099	1.2	0.300	3.5
PreciControl AMH 2	42.0	0.392	0.9	1.43	3.4

Method comparison

a) A comparison of the Elecsys AMH assay (y) with a commercially available AMH assay (x) using clinical samples gave the following correlations (ng/mL):

Number of samples measured: 548

Passing/Bablok[30]	Linear regression
y = 0.639x + 0.247	y = 0.592x + 0.541
τ = 0.919	r = 0.976

The sample concentrations were between 0.046 and 16.5 ng/mL.

b) A comparison of the Elecsys AMH assay (y) with a commercially available AMH assay (x) using clinical samples in the normal range up to 4 ng/mL gave the following correlations (ng/mL):

Number of samples measured: 340

Passing/Bablok[30]	Linear regression
y = 0.788x + 0.095	y = 0.720x + 0.206
τ = 0.884	r = 0.966

The sample concentrations were between 0.046 and 3.99 ng/mL.

Analytical specificity

The monoclonal antibodies used are highly specific to human AMH. The following cross-reactivities were found:

**Cross-reactant**	**Concentration tested**	**Cross-reactivity %**
Inhibin A	100 ng/mL	n. d.[c)]
Activin A	100 ng/mL	n. d.
LH	500 mIU/mL	n. d.
FSH	500 mIU/mL	n. d.

c) n. d. = not detectable

cobas®

References

1 Wilson CA, di Clemente N, Ehrenfels C, et al. Mullerian inhibiting substance requires its N-terminal domain for maintenance of biological activity, a novel finding within the transforming growth factor-beta superfamily. Mol Endocrinol 1993;7(2):247-257.

2 di Clemente N, Jamin SP, Lugovskoy A, et al. Processing of anti-mullerian hormone regulates receptor activation by a mechanism distinct from TGF-beta. Mol Endocrinol 2010;24(11):2193-206.

3 Grinspon RP, Rey RA. Anti-müllerian hormone and sertoli cell function in paediatric male hypogonadism. Horm Res Paediatr 2010;73(2):81-92.

4 Broekmans FJ, Visser JA, Laven JS, et al. Anti-Müllerian hormone and ovarian dysfunction. Trends Endocrinol Metab 2008;19(9):340-347.

5 Visser JA, de Jong FH, Laven JS, et al. Anti-Müllerian hormone: a new marker for ovarian function. Reproduction 2006;131(1):1-9.

6 Visser JA, Schipper I, Laven JS, et al. Anti-Müllerian hormone: an ovarian reserve marker in primary ovarian insufficiency. Nat Rev Endocrinol 2012;8(6):331-341.

7 Kelsey TW, Anderson RA, Wright P, et al. Data-driven assessment of the human ovarian reserve. Mol Hum Reprod 2012;18(2):79-87.

8 Nelson SM, Iliodromiti S, Fleming R, et al. Reference range for the antimüllerian hormone Generation II assay: a population study of 10,984 women, with comparison to the established Diagnostics Systems Laboratory nomogram. Fertil Steril 2014;101(2):523-529.

9 Tsepelidis S, Devreker F, Demeestere I, et al. Stable serum levels of anti-Mullerian hormone during the menstrual cycle: a prospective study in normo-ovulatory women. Hum Reprod 2007;22:1837–1840.

10 van Disseldorp J, Lambalk CB, Kwee J, et al. Comparison of inter- and intra-cycle variability of anti-Mullerian hormone and antral follicle counts. Hum Reprod 2010;25(1):221-227.

11 Overbeek A, Broekmans FJ, Hehenkamp WJ, et al. Intra-cycle fluctuations of anti-Müllerian hormone in normal women with a regular cycle: a re-analysis. Reprod Biomed Online 2012;24(6):664-669.

12 Kallio S, Puurunen J, Ruokonen A, et al. Antimüllerian hormone levels decrease in women using combined contraception independently of administration route. Fertil Steril 2013;99(5):1305-1310.

13 Nelson SM. Biomarkers of ovarian response: current and future applications. Fertil Steril 2013;99(4):963-969.

14 Anderson RA, Wallace WH. Antimüllerian hormone, the assessment of the ovarian reserve, and the reproductive outcome of the young patient with cancer. FertilSteril 2013;99(6):1469-1475.

15 Dewailly D, Andersen CY, Balen A, et al. The physiology and clinical utility of anti-Mullerian hormone in women. Hum Reprod Update 2014;20(3):370-385.

16 La Marca A, Sunkara SK. Individualization of controlled ovarian stimulation in IVF using ovarian reserve markers: from theory to practice. Hum Reprod Update 2014;20(1):124-140.

17 Josso N, Rey R, Picard JY. Testicular anti-Müllerian hormone: clinical applications in DSD. Semin Reprod Med 2012;30(5):364-373.

18 Rey RA, Grinspon RP, Gottlieb S, et al. Male hypogonadism: an extended classification based on a developmental, endocrine physiology-based approach. Andrology 2013;1(1):3-16.

19 La Marca A, Volpe A. The Anti-Mullerian hormone and ovarian cancer. Hum Reprod Update 2007;13(3):265-273.

20 Geerts I, Vergote I, Neven P, et al. The role of inhibins B and antimüllerian hormone for diagnosis and follow-up of granulosa cell tumors. Int J Gynecol Cancer 2009;19(5):847-855.

21 Dewailly D, Gronier H, Poncelet E, et al. Diagnosis of polycystic ovary syndrome (PCOS): revisiting the threshold values of follicle count on ultrasound and of the serum AMH level for the definition of polycystic ovaries. Hum Reprod 2011;26(11):3123-3129.

22 Iliodromiti S, Kelsey TW, Anderson RA, et al. Can anti-Mullerian hormone predict the diagnosis of polycystic ovary syndrome? A systematic review and meta-analysis of extracted data. J Clin Endocrinol Metab 2013;98(8):3332-3340.

23 Tehrani FR, Solaymani-Dodaran M, Tohidi M, et al. Modeling age at menopause using serum concentration of anti-mullerian hormone. J Clin Endocrinol Metab 2013;98(2):729-735.

24 Dólleman M, Depmann M, Eijkemans MJ, et al. Anti-Mullerian hormone is a more accurate predictor of individual time to menopause than mother's age at menopause. Hum Reprod 2014;29(3):584-591.

25 Rotterdam ESHRE/ASRM-Sponsored PCOS consensus workshop group. Revised 2003 consensus on diagnostic criteria and long-term health risks related to polycystic ovary syndrome (PCOS). Hum Reprod 2004;19(1):41-47.

26 Ferraretti AP, La Marca A, Fauser BC, et al. ESHRE working group on Poor Ovarian Response Definition. ESHRE consensus on the definition of 'poor response' to ovarian stimulation for in vitro fertilization: the Bologna criteria. Hum Reprod 2011;26(7):1616-1624.

27 van Tilborg TC, Eijkemans MJ, Laven JS, et al. The OPTIMIST study: optimisation of cost effectiveness through individualised FSH stimulation dosages for IVF treatment. A randomised controlled trial. BMC Womens Health 2012;18;12:29.

28 Nelson SM, Yates RW, Fleming R. Serum anti-Müllerian hormone and FSH: prediction of live birth and extremes of response in stimulated cycles--implications for individualization of therapy. Hum Reprod 2007;22(9):2414-21.

29 Arce JC, Andersen AN, Fernández-Sánchez M, et al. Ovarian response to recombinant human follicle-stimulating hormone: a randomized, antimüllerian hormone-stratified, dose-response trial in women undergoing in vitro fertilization/intracytoplasmic sperm injection. Fertil Steril 2014;102(6):1633-40.e5.

30 Bablok W, Passing H, Bender R, et al. A general regression procedure for method transformation. Application of linear regression procedures for method comparison studies in clinical chemistry, Part III. J Clin Chem Clin Biochem 1988 Nov;26(11):783-790.

For further information, please refer to the appropriate operator's manual for the analyzer concerned, the respective application sheets, the product information and the Method Sheets of all necessary components (if available in your country).

A point (period/stop) is always used in this Method Sheet as the decimal separator to mark the border between the integral and the fractional parts of a decimal numeral. Separators for thousands are not used.

Symbols

Roche Diagnostics uses the following symbols and signs in addition to those listed in the ISO 15223-1 standard:

CONTENT	Contents of kit
SYSTEM	Analyzers/Instruments on which reagents can be used
REAGENT	Reagent
CALIBRATOR	Calibrator
→	Volume after reconstitution or mixing
GTIN	Global Trade Item Number

COBAS, COBAS E, ELECSYS and PRECICONTROL are trademarks of Roche. INTRALIPID is a trademark of Fresenius Kabi AB.

All other product names and trademarks are the property of their respective owners.

Additions, deletions or changes are indicated by a change bar in the margin.

© 2016, Roche Diagnostics

CE

Roche Diagnostics GmbH, Sandhofer Strasse 116, D-68305 Mannheim
www.roche.com

Roche
