# Supplementary material for: Diagnostic accuracy of AMH for primary ovarian insufficiency/premature ovarian failure: a real-world cohort study
Source: Front Endocrinol (Lausanne). 2026 Feb 11;17:1742145. doi: 10.3389/fendo.2026.1742145 (PMC12932242; doi:10.3389/fendo.2026.1742145)
Supplement: Supplementary file 1 [file DataSheet1.zip › Quality Control Certificates/Value Sheet.PreciControl AMH-2017-08.pdf]

PreciControl AMH

cobas®

REF **06709966** 190

LOT **158473** — **2017-08**

		PreciControl AMH1 LOT **152549** *			PreciControl AMH2 LOT **152551** *			
Components	Method	Value	Range	1SD	Value	Range	1SD	Units
								Elecsys 2010 and **cobas e** 411 analyzers
**AMH**	Elecsys AMH **06331076**	0.95<br>6.78	0.75 - 1.15<br>5.36 - 8.20	0.07<br>0.47	4.79<br>34.2	4.07 - 5.51<br>29.1 - 39.3	0.24<br>1.71	ng/mL<br>pmol/L
**AMH**	Elecsys AMH **06331076** LOT **188352**	0.88<br>6.28	0.70 - 1.06<br>4.96 - 7.60	0.06<br>0.44	4.79<br>34.2	4.07 - 5.51<br>29.1 - 39.3	0.24<br>1.71	ng/mL<br>pmol/L
								MODULAR ANALYTICS E170, **cobas e** 601 and **cobas e** 602 analyzers
**AMH**	Elecsys AMH **06331076**	1.03<br>7.35	0.81 - 1.25<br>5.81 - 8.89	0.07<br>0.51	5.16<br>36.8	4.39 - 5.93<br>31.3 - 42.3	0.26<br>1.84	ng/mL<br>pmol/L
**AMH**	Elecsys AMH **06331076** LOT **188352**	0.97<br>6.93	0.77 - 1.17<br>5.47 - 8.39	0.07<br>0.49	4.90<br>35.0	4.17 - 5.64<br>29.8 - 40.3	0.25<br>1.75	ng/mL<br>pmol/L
**AMH**	Elecsys AMH **06331076** LOT **187139**	0.95<br>6.78	0.75 - 1.15<br>5.36 - 8.20	0.07<br>0.47	4.78<br>34.1	4.06 - 5.50<br>29.0 - 39.2	0.24<br>1.71	ng/mL<br>pmol/L

* The controls are not barcode-labeled and therefore must be run as external controls. All values and ranges must be entered manually.

Die Kontrollen sind nicht mit Barcode-Etiketten versehen und müssen deshalb als Fremdkontrollen vermessen werden. Alle Werte und Bereiche müssen manuell eingegeben werden.

Les contrôles n'ont pas de code-barres et doivent être dosés comme des contrôles externes. Tous les intervalles et valeurs doivent être saisis manuellement.

Los controles no tienen código de barras y deben tratarse como controles externos. Entonces, todos los valores e intervalos deben introducirse manualmente.

I controlli non hanno etichette con codice a barre e devono quindi essere eseguiti come controlli esterni. Tutti i valori e gli intervalli devono essere introdotti manualmente.

Os controlos não dispõem de rótulos de código de barras e, por isso, têm de ser utilizados como controlos externos. Todos os valores e intervalos têm de ser introduzidos manualmente.

Kontrollerne er ikke barkodemærket og skal derfor køres som eksterne kontroller. Alle værdier og områder skal indtastes manuelt.

Kontrollerna är inte streckkodsmärkta och måste därför köras på samma sätt som externa kontroller. Alla värden och intervall måste skrivas in manuellt.

Kontrollene er ikke strekkodemerkede og skal derfor analyseres som eksterne kontroller. Alle verdiene og aksepterte grense for avvik legges inn manuelt.

Kontroly nejsou označeny čárovými kódy a proto se musí měřit jako externí kontroly. Všechny hodnoty a rozmezí je nutné zadávat ručně.

Kontroly nie sú označené čiarovým kódom, preto musia byť zmerané ako externé kontroly. Všetky hodnoty a rozsahy musia byť vložené manuálne.

Kontrole nie posiadają etykiet z kodem kreskowym, w związku z czym należy oznaczać je jako kontrole zewnętrzne. Wszystkie wartości kontrolne i zakresy należy wprowadzić manualnie.

A kontrollok nincsenek vonalkóddal ellátva, ezért azokat külső kontrollként kell mérni. Az összes célértéket és -tartományt manuálisan kell megadni.

Τα διαλύματα ελέγχου δεν φέρουν ετικέτα με γραμμικό κώδικα και ως εκ τούτου πρέπει να αναλύονται ως εξωτερικά διαλύματα ελέγχου. Όλες οι τιμές και τα εύρη τιμών πρέπει να καταχωρηθούν από το χειριστή.

Kontroller barkod ile işaretli değildir ve bu nedenle dış kontrol olarak çalışılmalıdır. Tüm değerler ve aralıklar manuel olarak girilmelidir.

Контролите нямат етикети с баркод и затова трябва да се пускат като външни контроли. Всички стойности и обхвати трябва да се въведат ръчно.

Kontrollmaterjalid ei ole vöötkoodiga märgistatud ja neid tuleb seega analüüsida nagu väliseid kontrollmaterjale. Kõik väärtused ja vahemikud tuleb sisestada käsitsi.

Kontrolinė medžiaga nepažymėta brūkšniniu kodu, todėl turi būti tiriama kaip išorinė. Visos tikslinės reikšmės ir ribos turi būti įvestos rankiniu būdu.

Kontrolmateriāliem nav svītrkoda uzlīmju, tāpēc tie jāapstrādā kā ārējie kontrolmateriāli. Visas vērtības un diapazoni jāievada manuāli.

For translations, see glossary at the end of this document. / Übersetzungen siehe Glossar am Ende des Dokumentes. / Pour connaître les traductions, consulter le glossaire à la fin de ce document. / Para las traducciones consulte el glosario al final de este documento. / Per le traduzioni, vedere il glossario alla fine del presente documento. / Para traduções, ver glossário no fim deste documento. / Vedr. oversættelse - se gloseliste i slutningen af dette dokument. / Se ordlistan i slutet av detta dokument för översättningar. / Oversettelser, se den forklarende ordliste bakerst i dette dokumentet. / Překlady naleznete v glosáři na konci tohoto dokumentu. / Preklad pozri v glosári na konci tohto dokumentu. / Tłumaczenie, patrz słowniczek na końcu dokumentu. / A fordításokat lásd a dokumentum végén található fogalomtárban. / Για μεταφράσεις, δείτε το γλωσσάριο στο τέλος αυτού του εγγράφου. / Çeviriler için bu belgenin sonundaki sözlüğe bakınız.. / За превод, вижте речника в края на този документ. / Tõlgete koht vt käesoleva dokumendi lõpus olevat sõnastikku. / Dėl vertimų žr. šio dokumento pabaigoje esantį glosarijų. / Lai veiktu tulkošanu, skatiet glosāriju šī dokumenta beigās. / Для перевода см. глоссарий в конце этого документа. / Để dịch, vui lòng xem bảng chú giải thuật ngữ ở cuối tài liệu này.

PreciControl AMH

cobas®

REF 06709966 190 LOT 158473 2017-08

Контрольные материалы не маркированы штрих-кодом и должны использоваться как внешние контроли. Все значения и диапазоны должны быть внесены в анализатор вручную.

Glossary:

Kit / Kit / Coffret / Estuche / Confezione / Dispositivo / Kit / Kit / Kit / Souprava / Súprava / Zestaw / Csomag / Кıт / Kit / Кит / Komplekt / Rinkinys / Komplekts / Набор

Bottle / Flasche / Flacon / Frasco / Flacone / Frasco / Flaske / Flaska / Flaske / Nádobka / Fľaška / Butelka / Fiola / Φιάλη / Şişe / Шише / Pudel / Buteliukas / Pudele / Флакон

Components / Bestandteile / Constituant / Componente / Componenti / Componente / Komponenter / Komponenter / Komponenter / Složka / Zložky / Składniki / Összetevők / Συστατικά / Bileşenler / Компоненти / Koostisosad / Komponentai / Sastāvdaļas / Компоненты

Method / Methode / Méthode / Método / Metodo / Método / Metode / Metod / Metode / Metoda / Metóda / Metoda / Módszer / Μέθοδος / Yöntem / Метод / Meetod / Metodas / Metode / Метод

Value / Wert / Valeur / Valor / Valore / Valor / Værdi / Värde / Verdi / Hodnota / Hodnota / Wartość / Érték / Τιμή / Değer / Стойност / Väärtus / Vertė / Vērtība / Значение

Range / Bereich / Intervalle / Intervalo / Intervallo / Intervalo / Område / Intervall / Område / Rozsah / Rozsah / Zakres / Tartomány / Εύρος / Aralık / Обхват / Vahemik / Intervalas / Diapazons / Диапазон

1SD / 1SD / 1s / 1DE / 1DS / 1DP / 1SD / 1SD / 1SD / 1SD / 1SD / 1SD / 1SD / 1SD / 1SD / 1SD / 1SD / 1SD / 1SN / 1SD

Units / Maßeinheit / Unité / Unidad / Unità di misura / Unidade / Enhed / Enhet / Enhet / Jednotka / Jednotka / Jednostka / Mértékegység / Μονάδα / Birim / Единица / Ühik / Vienetas / Vienība / Единица

© 2016, Roche Diagnostics

Roche Diagnostics GmbH, Sandhofer Strasse 116, D-68305 Mannheim
www.roche.com
