# Supplementary material for: Diagnostic accuracy of AMH for primary ovarian insufficiency/premature ovarian failure: a real-world cohort study
Source: Front Endocrinol (Lausanne). 2026 Feb 11;17:1742145. doi: 10.3389/fendo.2026.1742145 (PMC12932242; doi:10.3389/fendo.2026.1742145)
Supplement: Supplementary file 1 [file DataSheet1.zip › Quality Control Certificates/Value Sheet.PreciControl AMH-2018-03.pdf]

1 / 2 83.2% Tools Sign Comme

vs_06709966190V9.0

PreciControl AMH

REF 06709966 190

LOT 220253

cobas®

2018-03

		PreciControl AMH1 LOT 201715 *			PreciControl AMH2 LOT 201716 *			
Components	Method	Value	Range	1SD	Value	Range	1SD	Units
								**cobas e 411 analyzer**
AMH	Elecsys AMH	1.08	0.85 - 1.31	0.08	5.47	4.65 - 6.29	0.27	ng/mL
	06331076	7.71	6.09 - 9.33	0.54	39.1	33.2 - 45.0	1.96	pmol/L
								MODULAR ANALYTICS E170, **cobas e** 601 and **cobas e** 602 analyzers
AMH	Elecsys AMH	1.08	0.85 - 1.31	0.08	5.47	4.65 - 6.29	0.27	ng/mL
	06331076	7.71	6.09 - 9.33	0.54	39.1	33.2 - 45.0	1.96	pmol/L

* The controls are not barcode-labeled and therefore must be run as external controls. All values and ranges must be entered manually.

Die Kontrollen sind nicht mit Barcode-Etiketten versehen und müssen deshalb als Fremdkontrollen vermessen werden. Alle Werte und Bereiche müssen manuell eingegeben werden.

Les contrôles n'ont pas de code-barres et doivent être dosés comme des contrôles externes. Tous les intervalles et valeurs doivent être saisis manuellement.

Los controles no tienen codigo de barras y deben tratarse como controles externos. Entonces, todos los valores e intervalos deben introducirse manualmente.
