# Supplementary material for: Diagnostic accuracy of AMH for primary ovarian insufficiency/premature ovarian failure: a real-world cohort study
Source: Front Endocrinol (Lausanne). 2026 Feb 11;17:1742145. doi: 10.3389/fendo.2026.1742145 (PMC12932242; doi:10.3389/fendo.2026.1742145)
Supplement: Supplementary file 1 [file DataSheet1.zip › Quality Control Certificates/Value Sheet.PreciControl AMH-2025-04.pdf]

07957211500V5.0

# PreciControl AMH Plus

cobas®

REF 07957211190 | 07957211500 | → **4 x 2.0 mL**

## English

### Intended use

PreciControl AMH Plus is used for quality control of the Elecsys AMH Plus immunoassay on the Elecsys and **cobas e** immunoassay analyzers.

### Summary

PreciControl AMH Plus is a lyophilized control serum based on an equine serum matrix with added bovine AMH (male fetal bovine serum) in 2 concentration ranges.

The controls are used for monitoring the accuracy and precision of the Elecsys AMH Plus immunoassay.

### Reagents - working solutions

- PC AMH Plus 1: 2 bottles, each for 2.0 mL of control serum
- PC AMH Plus 2: 2 bottles, each for 2.0 mL of control serum

AMH in 2 concentration ranges (approximately 7 pmol/L or 1 ng/mL and approximately 35 pmol/L or 5 ng/mL ) in an equine serum matrix; preservative.

### Target values and ranges

The target values and ranges were determined and evaluated by Roche. They were obtained using the Elecsys AMH Plus assay reagents and analyzers available at the time of testing.

The controls will be handled automatically by the **cobas e** 402, **cobas e** 602 and **cobas e** 801 analyzers.

The target values and ranges (original and updated) and the value sheet are available electronically via the **cobas** link.

**cobas e** 411 and **cobas e** 601 analyzers: The lot-specific value sheet is included in the control or reagent kit and is also provided electronically via the **cobas** link. The controls are not barcode labeled and therefore have to be run like external controls. All values and ranges have to be entered manually. Please refer to the section “QC” in the operator's manual or to the online help of the instrument software.

Non-barcode labeled controls: Only one target value and range for each control level can be entered in the analyzer. The reagent lot-specific target values must be re-entered each time when a specific reagent lot with different control target values and ranges is used. Two reagent lots with different control target values and ranges cannot be used in parallel in the same run.

Please make sure that the correct values are used.

If the target values and control ranges are updated, this information is conveyed in an additional value sheet included in the reagent kit. This value sheet lists all control lots to which the new values apply. If some of the values remain unchanged, the original values and the original value sheet included in the control kit remain valid.

Results must be within the specified ranges. In the event that increasing or decreasing trends, or any other suddenly occurring deviations beyond the range limits are observed, all test steps must be checked.

Traceability information is given in the Method Sheet of the relevant Elecsys assay.

Each laboratory should establish corrective measures to be taken if values fall outside the defined limits.

### Precautions and warnings

For in vitro diagnostic use.
Exercise the normal precautions required for handling all laboratory reagents.
Disposal of all waste material should be in accordance with local guidelines.
Safety data sheet available for professional user on request.

This kit contains components classified as follows in accordance with the Regulation (EC) No. 1272/2008:

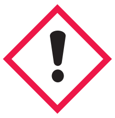

Warning

H317 May cause an allergic skin reaction.

H412 Harmful to aquatic life with long lasting effects.

**Prevention:**

P261 Avoid breathing dust.

P273 Avoid release to the environment.

P280 Wear protective gloves.

**Response:**

P333 + P313 If skin irritation or rash occurs: Get medical advice/attention.

P362 + P364 Take off contaminated clothing and wash it before reuse.

**Disposal:**

P501 Dispose of contents/container to an approved waste disposal plant.

**Hazardous components:**

- 2-methyl-2H-isothiazol-3-one hydrochloride

Product safety labeling follows EU GHS guidance.

Contact phone: all countries: +49-621-7590

The controls may not be used after the expiration date.

Avoid foam formation in all reagents and sample types (specimens, calibrators and controls).

### Handling

Carefully dissolve the contents of 1 bottle by adding exactly 2.0 mL of distilled or deionized water and allow to stand closed for 15 minutes to reconstitute. Mix carefully, avoiding foam formation.

When measuring non-barcoded controls, use only recommended sample tubes, “cup on tube” or “cup on rack”.

Transfer aliquots of the reconstituted control into appropriate tubes. Aliquots intended for storage at -20 °C (± 5 °C) should be frozen immediately.

Perform **only one** control procedure per aliquot.

Please note: Both the vial labels and the additional labels (if available) contain a barcode for the **cobas e** 402, **cobas e** 602 and **cobas e** 801 analyzers only. Place the vial on the analyzer as usual.

### Storage and stability

Store at 2-8 °C.

The lyophilized control serum is stable up to the stated expiration date.

| Stability of the reconstituted control serum: | |
|---|---|
| at -20 °C (± 5 °C) | 28 days (freeze only once) |
| on the analyzers at 20-25 °C | use only once |

Store controls **upright** in order to prevent the control solution from adhering to the snap-cap.

### Materials provided

- PreciControl AMH Plus, 2 x 2 empty labeled snap-cap bottles, 2 x 6 bottle labels

### Materials required (but not provided)

- **cobas e** immunoassay analyzers and assay reagents
- Distilled or deionized water

See the assay Method Sheet and the operator’s manual for additionally required materials.

### Assay

Treat the reconstituted control serum in the system-compatible labeled bottles for analysis in the same way as patient samples.

Ensure the controls are at 20-25 °C prior to measurement.

Run controls daily in parallel with patient samples, once per reagent kit, and whenever a calibration is performed. The control intervals and limits should be adapted to each laboratory’s individual requirements.

# PreciControl AMH Plus

Follow the applicable government regulations and local guidelines for quality control.

For further information, please refer to the appropriate user guide or operator’s manual for the analyzer concerned, the respective application sheets and the Method Sheets of all necessary components (if available in your country).

A point (period/stop) is always used in this Method Sheet as the decimal separator to mark the border between the integral and the fractional parts of a decimal numeral. Separators for thousands are not used.

**Symbols**

Roche Diagnostics uses the following symbols and signs in addition to those listed in the ISO 15223-1 standard:

| | |
|---|---|
| CONTENT | Contents of kit |
| SYSTEM | Analyzers/Instruments on which reagents can be used |
| REAGENT | Reagent |
| CALIBRATOR | Calibrator |
| → | Volume for reconstitution |
| GTIN | Global Trade Item Number |
| Rx only | For USA: Caution: Federal law restricts this device to sale by or on the order of a physician. |

COBAS, ELECSYS, NAVIFY and PRECICONTROL are trademarks of Roche.

All other product names and trademarks are the property of their respective owners.

Additions, deletions or changes are indicated by a change bar in the margin.

© 2024, Roche Diagnostics

Roche Diagnostics GmbH
Sandhofer Strasse 116
68305 Mannheim, Germany
www.roche.com

+800 5505 6606
