# Supplementary material for: Diagnostic accuracy of AMH for primary ovarian insufficiency/premature ovarian failure: a real-world cohort study
Source: Front Endocrinol (Lausanne). 2026 Feb 11;17:1742145. doi: 10.3389/fendo.2026.1742145 (PMC12932242; doi:10.3389/fendo.2026.1742145)
Supplement: Supplementary file 1 [file DataSheet1.zip › Quality Control Certificates/广东省中医院检验科ISO书2025-2029.pdf]

Name: Clinical Laboratory of Guangdong Hospital of Traditional Chinese Medicine

Address: No.111, Dade Road, Yuexiu District, Guangzhou, Guangdong, China

Registration No. CNAS MT0003

Accreditation Criteria: ISO 15189:2022 and relevant requirements of CNAS

CHINA NATIONAL ACCREDITATION SERVICE FOR CONFORMITY ASSESSMENT

Effective Date: 2025-03-19 Expiry Date: 2029-02-05

SCHEDULE OF ACCREDITATION CERTIFICATE

SCHEDULE 2 ACCREDITED EXAMINATION SCOPE

№	Examination Item	Sample Type	Analytical Method	Note	Effective Date
A Laboratory Medicine					
AA Clinical Hematology					
1	White blood cell count(WBC)	Whole blood	Flow cytometry and nucleic acid fluorescein staining		2025-03-19
			Flow cytometry and nucleic acid fluorescein staining		
			Flow cytometry and nucleic acid fluorescein staining		
			Flow cytometry and nucleic acid fluorescein staining		
			Flow cytometry and nucleic acid fluorescein staining		
			Flow cytometry and nucleic acid fluorescein staining		

中国合格评定国家认可委员会
认可证书专用章

在线扫码获取验证

The scope of the accreditation in Chinese remains the definitive version.

CHINA NATIONAL ACCREDITATION SERVICE FOR CONFORMITY ASSESSMENT
SCHEDULE OF ACCREDITATION CERTIFICATE

№	Examination Item	Sample Type	Analytical Method	Note	Effective Date
2	Platelet count(PLT)	Whole blood	Sheath flow impedance		2025-03-19
			Sheath flow impedance		
			Sheath flow impedance		
			Sheath flow impedance		
			Sheath flow impedance		
			Sheath flow impedance		
3	Red blood cell count(RBC)	Whole blood	Sheath flow impedance		2025-03-19
			Sheath flow impedance		
			Sheath flow impedance		
			Sheath flow impedance		
			Sheath flow impedance		
			Sheath flow impedance		
4	Hemoglobin(HGB)	Whole blood	Sodium Lauryl Sulfate Hemoglobin（SLS-Hgb）		2025-03-19
			Sodium Lauryl Sulfate Hemoglobin（SLS-Hgb）		

中国合格评定国家认可委员会 认可证书专用章

在线扫码获取验证

The scope of the accreditation in Chinese remains the definitive version.

CHINA NATIONAL ACCREDITATION SERVICE FOR CONFORMITY ASSESSMENT
SCHEDULE OF ACCREDITATION CERTIFICATE

№	Examination Item	Sample Type	Analytical Method	Note	Effective Date
			Sodium Lauryl Sulfate Hemoglobin（SLS-Hgb）		
			Sodium Lauryl Sulfate Hemoglobin（SLS-Hgb）		
			Sodium Lauryl Sulfate Hemoglobin（SLS-Hgb）		
			Sodium Lauryl Sulfate Hemoglobin（SLS-Hgb）		
5	Hematocrit(Hct)	Whole blood	calculation		2025-03-19
			calculation		
			calculation		
			calculation		
			calculation		
			Calculation method		
6	Mean corpuscular volume(MCV)	Whole blood	Sheath flow impedance		2025-03-19
			Sheath flow impedance		
			Sheath flow impedance		
			Sheath flow impedance		

中国合格评定国家认可委员会
认可证书专用章

在线扫码获取验证

The scope of the accreditation in Chinese remains the definitive version.

CHINA NATIONAL ACCREDITATION SERVICE FOR CONFORMITY ASSESSMENT
SCHEDULE OF ACCREDITATION CERTIFICATE

№	Examination Item	Sample Type	Analytical Method	Note	Effective Date
			Sheath flow impedance		
			Sheath flow impedance		
7	Mean corpuscIar hemoglobin(MCH)	Whole blood	calculation		2025-03-19
			calculation		
			calculation		
			calculation		
			calculation		
			Calculation method		
8	Mean corpuscIar hemoglobin concentration(MCHC)	Whole blood	calculation		2025-03-19
			calculation		
			calculation		
			calculation		
			calculation		
			Calculation method		

中国合格评定国家认可委员会
认可证书专用章
在线扫码获取验证

The scope of the accreditation in Chinese remains the definitive version.

№	Examination Item	Sample Type	Analytical Method	Note	Effective Date
9	Leukocyte differential count（DC）	Whole blood	Flow cytometry and nucleic acid fluorescein staining		2025-03-19
			Flow cytometry and nucleic acid fluorescein staining		
			Flow cytometry and nucleic acid fluorescein staining		
			Flow cytometry and nucleic acid fluorescein staining		
			Flow cytometry and nucleic acid fluorescein staining		
			Flow cytometry and nucleic acid fluorescein staining		
10	Morphology evaluation，peripheral blood smear	Whole blood	Microscopic examination		2025-03-19
			Microscopic examination		
11	Erythrocyte sedimentation rate（ESR）	Whole blood	capillary method		2025-03-19
			capillary method		
			capillary method		
12	Prothrombin time（PT）	plasma	Coagulation assay		2025-03-19
			Coagulation assay		
			Coagulation assay		

CHINA NATIONAL ACCREDITATION SERVICE FOR CONFORMITY ASSESSMENT
SCHEDULE OF ACCREDITATION CERTIFICATE
中国合格评定国家认可委员会
认可证书专用章
在线扫码获取验证

The scope of the accreditation in Chinese remains the definitive version.

№	Examination Item	Sample Type	Analytical Method	Note	Effective Date
13	Activated partial thromboplastin time（APTT）	plasma	Coagulation assay<br>Coagulation assay<br>Coagulation assay		2025-03-19
14	Fibrinogen（Fg）	plasma	Thrombin Coagulation assay（Clauss）<br>Thrombin Coagulation assay（Clauss）<br>Thrombin Coagulation assay（Clauss）		2025-03-19
15	Thrombin time（TT）	plasma	Coagulation assay<br>Coagulation assay<br>Coagulation assay		2025-03-19
16	D-dimer （DD	plasma	Immunoturbidimetric assay<br>Immunoturbidimetric assay<br>Immunoturbidimetric assay		2025-03-19
17	Fibrin（ogen） degradation products （FDP）	plasma	Immunoturbidimetric assay<br>Immunoturbidimetric assay		2025-03-19

CHINA NATIONAL ACCREDITATION SERVICE FOR CONFORMITY ASSESSMENT
SCHEDULE OF ACCREDITATION CERTIFICATE
中国合格评定国家认可委员会
认可证书专用章
在线扫码获取验证

The scope of the accreditation in Chinese remains the definitive version.

№	Examination Item	Sample Type	Analytical Method	Note	Effective Date
			Immunoturbidimetric assay		
18	Bone marrow smear cytology	Bone Marrow	Microscopic examination		2025-03-19
AB Body Fluid examination					
19	Urine color	urine	Photoelectric colorimetry		2025-03-19
			Photoelectric colorimetry		
			Human eye observation		
20	Urine turbidity	urine	Scattering method		2025-03-19
			Scattering method		
			Human eye observation		
21	pH	urine	Photoelectric colorimetry		2025-03-19
			Photoelectric colorimetry		
22	Specific gravity	urine	Refraction method		2025-03-19
			Refraction method		
23	Protein （PRO	urine	Photoelectric colorimetry		2025-03-19

CHINA NATIONAL ACCREDITATION SERVICE FOR CONFORMITY ASSESSMENT
SCHEDULE OF ACCREDITATION CERTIFICATE
中国合格评定国家认可委员会
认可证书专用章
在线扫码获取验证

The scope of the accreditation in Chinese remains the definitive version.

№	Examination Item	Sample Type	Analytical Method	Note	Effective Date
			Photoelectric colorimetry		
24	Glucose （GLU）	urine	Photoelectric colorimetry		2025-03-19
			Photoelectric colorimetry		
25	Acetone body	urine	Photoelectric colorimetry		2025-03-19
			Photoelectric colorimetry		
26	Bilirubin （BIL）	urine	Photoelectric colorimetry		2025-03-19
			Photoelectric colorimetry		
27	Urobilinogen （UBG）	urine	Photoelectric colorimetry		2025-03-19
			Photoelectric colorimetry		
28	Urine nitrite test （NIT）	urine	Photoelectric colorimetry		2025-03-19
			Photoelectric colorimetry		
29	Leukocyte esterase	urine	Photoelectric colorimetry		2025-03-19
			Photoelectric colorimetry		
30	Occult blood or red cell	urine	Photoelectric colorimetry		2025-03-19

CHINA NATIONAL ACCREDITATION SERVICE FOR CONFORMITY ASSESSMENT
SCHEDULE OF ACCREDITATION CERTIFICATE
中国合格评定国家认可委员会 认可证书专用章
在线扫码获取验证

The scope of the accreditation in Chinese remains the definitive version.

№	Examination Item	Sample Type	Analytical Method	Note	Effective Date
			Photoelectric colorimetry		
31	Urine formed element	urine	auto-identification of microscope image		2025-03-19
			auto-identification of microscope image		
			Auto-identification Of Microscope Image		
			Auto-identification Of Microscope Image		
32	Examination of urinary sediments	urine	microscope examination		2025-03-19
33	Fecal physical examination	feces	Human eye observation		2025-03-19
			Instrument built-in microscope automatic scanning method		
34	Fecal formed element examination	feces	microscope examination		2025-03-19
			Instrument built-in microscope automatic scanning method		
			Instrument built-in microscope automatic scanning method		
35	Occult blood test （OBT）	feces	Colloidal gold method		2025-03-19
			Colloidal gold method		
			Colloidal gold method		

CHINA NATIONAL ACCREDITATION SERVICE FOR CONFORMITY ASSESSMENT
SCHEDULE OF ACCREDITATION CERTIFICATE
中国合格评定国家认可委员会
认可证书专用章
在线扫码获取验证

The scope of the accreditation in Chinese remains the definitive version.

CHINA NATIONAL ACCREDITATION SERVICE FOR CONFORMITY ASSESSMENT
SCHEDULE OF ACCREDITATION CERTIFICATE

№	Examination Item	Sample Type	Analytical Method	Note	Effective Date
36	Parasite eggs test	fecal	Microscope Examination		2025-03-19
			Instrument built-in microscope automatic scanning method		
			Instrument built-in microscope automatic scanning method		
AC Clinical Chemistry					
37	Creatine kinase（CK）	Serum	Rate		2025-03-19
		Serum	Rate		2025-03-19
		Plasma	Dry Chemistry		2025-03-19
		Plasma	Dry Chemistry		2025-03-19
38	Creatine kinase MB（CK-MB）	Serum	Immunosuppression		2025-03-19
		Serum	Immunosuppression		2025-03-19
		Plasma	Dry Chemistry		2025-03-19
		Plasma	Dry Chemistry		2025-03-19
39	Mass assay of creatine kinase isoenzyme (CK-MB mass)	plasma	Electrochemistry luminescence method		2025-03-19
		plasma	Electrochemistry luminescence method		2025-03-19

中国合格评定国家认可委员会
认可证书专用章

在线扫码获取验证

The scope of the accreditation in Chinese remains the definitive version.

CHINA NATIONAL ACCREDITATION SERVICE FOR CONFORMITY ASSESSMENT
SCHEDULE OF ACCREDITATION CERTIFICATE

№	Examination Item	Sample Type	Analytical Method	Note	Effective Date
40	Asparate aminotransferase（AST）	Serum	Rate		2025-03-19
		Serum	Rate		2025-03-19
		Plasma	Dry Chemistry		2025-03-19
		Plasma	Dry Chemistry		2025-03-19
41	Alanine aminotransferase（ALT）	Serum	Rate		2025-03-19
		Serum	Rate		2025-03-19
42	Lactate dehydrogenase（LDH）	Serum	Rate		2025-03-19
		Serum	Rate		2025-03-19
		Plasma	Dry Chemistry		2025-03-19
		Plasma	Dry Chemistry		2025-03-19
43	Alkaline phosphatase（ALP）	Serum	Rate		2025-03-19
		Serum	Rate		2025-03-19
44	Gamma glutamyl transpeptidase（GGT）	Serum	Rate		2025-03-19
		Serum	Rate		2025-03-19

中国合格评定国家认可委员会
认可证书专用章

在线扫码获取验证

The scope of the accreditation in Chinese remains the definitive version.

№	Examination Item	Sample Type	Analytical Method	Note	Effective Date
45	cholinesterase	Plasma			2025-03-19
46	Adenosine deaminase(ADA)	Serum	Peroxidase method		2025-03-19
		Serum	Peroxidase method		2025-03-19
47	Amylase（AMS）	Serum	EPS-G7		2025-03-19
		Plasma	Dry Chemistry		2025-03-19
48	Amylase（AMS）	Plasma			2025-03-19
49	Hydroxybutirate dehydrogenase	Serum	Rate		2025-03-19
		Serum	Rate		2025-03-19
50	Total protein（TP）	Serum	biuret method		2025-03-19
		Serum	biuret method		2025-03-19
51	Albumin（ALB）	Serum	bromcresol green method(BCG)		2025-03-19
		Serum	bromcresol green method(BCG)		2025-03-19
52	Creatinine（Cr）	Serum	Plus		2025-03-19
		Serum	Plus		2025-03-19

CHINA NATIONAL ACCREDITATION SERVICE FOR CONFORMITY ASSESSMENT
SCHEDULE OF ACCREDITATION CERTIFICATE
中国合格评定国家认可委员会
认可证书专用章
在线扫码获取验证

The scope of the accreditation in Chinese remains the definitive version.

№	Examination Item	Sample Type	Analytical Method	Note	Effective Date
		Plasma	Dry Chemistry		2025-03-19
		Plasma	Dry Chemistry		2025-03-19
53	Uric acid（UA）	Serum	Uricase		2025-03-19
		Serum	Uricase		2025-03-19
		Plasma	Dry Chemistry		2025-03-19
54	Urea	Serum	Urease /GLDH		2025-03-19
		Serum	Urease /GLDH		2025-03-19
		Plasma	Dry Chemistry		2025-03-19
55	Homocysteine（HCY）	Serum	Enzymatic cycling		2025-03-19
56	Prealbumine（PA）	Serum	Immunoneturbidimetric		2025-03-19
		Serum	Immunoneturbidimetric		2025-03-19
57	C reactive protein（CRP）	Serum	Immunoneturbidimetric		2025-03-19
			Immunoneturbidimetric		
58	β 2 microglubulin	Serum	Nephelometry		2025-03-19

CHINA NATIONAL ACCREDITATION SERVICE FOR CONFORMITY ASSESSMENT
SCHEDULE OF ACCREDITATION CERTIFICATE
中国合格评定国家认可委员会 认可证书专用章
在线扫码获取验证

The scope of the accreditation in Chinese remains the definitive version.

CHINA NATIONAL ACCREDITATION SERVICE FOR CONFORMITY ASSESSMENT
SCHEDULE OF ACCREDITATION CERTIFICATE

№	Examination Item	Sample Type	Analytical Method	Note	Effective Date
59	Transferritin（TFN）	Serum	Nephelometry		2025-03-19
60	Troponin T	Plasma	Electrochemiluminescence		2025-03-19
		Plasma	Electrochemiluminescence		2025-03-19
61	Myoglobin	Plasma	Electrochemiluminescence		2025-03-19
		Plasma	Electrochemiluminescence		2025-03-19
62	Type B natriuretic peptide（BNP）	Plasma	ChemiLuminescence		2025-03-19
63	N-terminal type B pronatriuretic peptide（NT-ProBNP）	Plasma	Electrochemiluminescence		2025-03-19
		Plasma	Electrochemiluminescence		2025-03-19
64	Ferritin	Serum	Chemiluminescent microparticle immunoassay		2025-03-19
65		Serum	Latex method		2025-03-19
		Whole blood	Immunoturbidimetry		2025-03-19
		Whole blood	Immunoturbidimetry		2025-03-19
		Whole blood	Immunoturbidimetry		2025-03-19
		Whole blood	Immunoturbidimetry		2025-03-19

中国合格评定国家认可委员会
认可证书专用章

在线扫码获取验证

The scope of the accreditation in Chinese remains the definitive version.

CHINA NATIONAL ACCREDITATION SERVICE FOR CONFORMITY ASSESSMENT
SCHEDULE OF ACCREDITATION CERTIFICATE

№	Examination Item	Sample Type	Analytical Method	Note	Effective Date
66	Glucose（GLU）	Serum	Glucose hexokinase		2025-03-19
		Serum	Glucose hexokinase		2025-03-19
		Plasma	Dry Chemistry		2025-03-19
		Plasma	Dry Chemistry		2025-03-19
		peripheral blood	Glucose Dehydrogen ase method		2025-03-19
67	Glycated hemoglobin A1c（HbA1c）	whole blood	Capillary electrophoresis		2025-03-19
		whole blood	Affinity chromatography		2025-03-19
68	Fructosamine（GSP）	Serum	Colorimetry		2025-03-19
69	Lactate（LAC）	plasma	Dry Chemistry		2025-03-19
70	β-hydroxybutyrate （b-HB）	plasma	β-hydroxybutyrate dehydrogenase method		2025-03-19
71	Triglyceride（TG）	Serum	GPO-PAP		2025-03-19
		Serum	GPO-PAP		2025-03-19
72	Total cholesterol（TC）	Serum	CHOD-PAP		2025-03-19
		Serum	CHOD-PAP		2025-03-19

中国合格评定国家认可委员会 认可证书专用章

在线扫码获取验证

The scope of the accreditation in Chinese remains the definitive version.

№	Examination Item	Sample Type	Analytical Method	Note	Effective Date
73	HDL-cholesterol（HDL-C）	Serum	Direct		2025-03-19
		Serum	Direct		2025-03-19
74	LDL-choleste rol（LDL-C）	Serum	Direct		2025-03-19
		Serum	Direct		2025-03-19
75	Total bile acids（TBA）	Serum	Enzymatic cycling		2025-03-19
		Serum	Enzymatic cycling		2025-03-19
76	Lipoprotein （a）（Lp（a））	Serum	Immunoneturbidimetric		2025-03-19
		Serum	Immunoneturbidimetric		2025-03-19
77	Apolipoprotein A1（ApoA1）	Serum	Immunoturbidimetry		2025-03-19
		Serum	Immunoturbidimetry		2025-03-19
78	Apolipoprotein B（ApoB）	Serum	Immunoturbidimetry		2025-03-19
		Serum	Immunoturbidimetry		2025-03-19
79	Vitamin B12	Serum	Chemiluminescent microparticle immunoassay		2025-03-19
80	Folic acid	Serum	Chemiluminescent microparticle immunoassay		2025-03-19

CHINA NATIONAL ACCREDITATION SERVICE FOR CONFORMITY ASSESSMENT
SCHEDULE OF ACCREDITATION CERTIFICATE
中国合格评定国家认可委员会 认可证书专用章
在线扫码获取验证

The scope of the accreditation in Chinese remains the definitive version.

№	Examination Item	Sample Type	Analytical Method	Note	Effective Date
81	Sodium（Na）	Serum	ISE-indirect		2025-03-19
		Serum	ISE-indirect		2025-03-19
		Serum	ISE-indirect		2025-03-19
		Plasma	Dry Chemistry		2025-03-19
		Plasma	Dry Chemistry		2025-03-19
82	Potassium（K）	Serum	ISE-indirect		2025-03-19
		Serum	ISE-indirect		2025-03-19
		Serum	ISE-indirect		2025-03-19
		Plasma	Dry Chemistry		2025-03-19
		Plasma	Dry Chemistry		2025-03-19
83	Chloride（Cl）	Serum	ISE-indirect		2025-03-19
		Serum	ISE-indirect		2025-03-19
		Serum	ISE-indirect		2025-03-19
		Plasma	Dry Chemistry		2025-03-19

CHINA NATIONAL ACCREDITATION SERVICE FOR CONFORMITY ASSESSMENT
SCHEDULE OF ACCREDITATION CERTIFICATE
中国合格评定国家认可委员会
认可证书专用章
在线扫码获取验证

The scope of the accreditation in Chinese remains the definitive version.

№	Examination Item	Sample Type	Analytical Method	Note	Effective Date
		Plasma	Dry Chemistry		2025-03-19
84	Magnesium （Mg）	Serum	Colorimetry		2025-03-19
		Serum	Colorimetry		2025-03-19
		Plasma	Dry Chemistry		2025-03-19
85	Calcium（Ca）	Serum	Colorimetry		2025-03-19
		Serum	Colorimetry		2025-03-19
		Plasma	Dry Chemistry		2025-03-19
		Plasma	Dry Chemistry		2025-03-19
86	Calcium，ionized	whole blood	Electrode-direct		2025-03-19
		whole blood	Electrode-direct		2025-03-19
87	Inorganic phosphate	Serum	Phosphomolybdate-UV		2025-03-19
		Serum	Phosphomolybdate-UV		2025-03-19
		Plasma	Dry Chemistry		2025-03-19
88	Arterial blood pH	whole blood	Electrode-direct		2025-03-19

ilac-MRA CNAS
CHINA NATIONAL ACCREDITATION SERVICE FOR CONFORMITY ASSESSMENT
SCHEDULE OF ACCREDITATION CERTIFICATE
中国合格评定国家认可委员会 认可证书专用章
在线扫码获取验证

The scope of the accreditation in Chinese remains the definitive version.

№	Examination Item	Sample Type	Analytical Method	Note	Effective Date
		whole blood	Electrode-direct		2025-03-19
89	Arterial blood carbon dioxide partial pressure	whole blood	Electrode-direct		2025-03-19
		whole blood	Electrode-direct		2025-03-19
90	Oxygen partial pressure	whole blood	Electrode-direct		2025-03-19
		whole blood	Electrode-direct		2025-03-19
91	Plasma bicarbonate concentration(TCO2)	Serum	Colorimetry		2025-03-19
		Serum	enzyme method		2025-03-19
		plasma	Dry Chemistry		2025-03-19
		plasma	Dry Chemistry		2025-03-19
92	Total Bilirubin （TBIL）	Serum	Vanadium acid oxidation method		2025-03-19
		Serum	Diazo		2025-03-19
93	Bilirubin， Conjugated （DBIL）	Serum	Vanadium acid oxidation method		2025-03-19
		Serum	Diazo		2025-03-19
94	Serum	Serum	Colorimetry		2025-03-19

CHINA NATIONAL ACCREDITATION SERVICE FOR CONFORMITY ASSESSMENT
SCHEDULE OF ACCREDITATION CERTIFICATE

The scope of the accreditation in Chinese remains the definitive version.

在线扫码获取验证

№	Examination Item	Sample Type	Analytical Method	Note	Effective Date
95	Unsaturated iron binding capacity	Serum	Colorimetry		2025-03-19
96	Digitoxin	Serum	ChemiLuminescence		2025-03-19
97	Theophylline	Serum	ChemiLuminescence		2025-03-19
98	Prolactin	Serum	Electrochemiluminescence		2025-03-19
99	Luteinizing Hormone （LH）	Serum	Electrochemiluminescence		2025-03-19
100	Follicle-stimu lating hormone （FSH）	Serum	Electrochemiluminescence		2025-03-19
101	Thyroid-stimulating hormone （TSH）	Serum	Direct Chemilum inescence method		2025-03-19
102	Triiodothyronine（T3）	Serum	Direct Chemilum inescence method		2025-03-19
103	Free triiodothyronine（FT3）	Serum	Direct Chemilum inescence method		2025-03-19
104	Thyroxine（T4）	Serum	Direct Chemilum inescence method		2025-03-19
105	Thyroxine（T4）	Serum	Direct Chemilum inescence method		2025-03-19
106	Parathyroid Hormone （PTH）	Serum	Chemiluminescence		2025-03-19
107	Procalcitonin（PCT）	Plasma	Electrochemiluminescence		2025-03-19
			Electrochemiluminescence		

CHINA NATIONAL ACCREDITATION SERVICE FOR CONFORMITY ASSESSMENT
SCHEDULE OF ACCREDITATION CERTIFICATE
中国合格评定国家认可委员会 认可证书专用章
在线扫码获取验证

The scope of the accreditation in Chinese remains the definitive version.

№	Examination Item	Sample Type	Analytical Method	Note	Effective Date
108	Cortisol	Serum	Direct Chemilum inescence method		2025-03-19
109	Estradiol （E2）	Serum	Electrochemiluminescence		2025-03-19
110	Progesterone （P）	Serum	Electrochemiluminescence		2025-03-19
			Electrochemiluminescence		
111	Testosterone （T）	Serum	Electrochemiluminescence		2025-03-19
112	Chorionic gonadotropin beta-subunit （β-HCG）	Serum	Electrochemiluminescence		2025-03-19
		Serum	Electrochemiluminescence		2025-03-19
113	AMH	Serum	Electrochemiluminescence		2025-03-19
114	Insulin	Serum	Direct Chemilum inescence method		2025-03-19
115	C-peptide	Serum	Direct Chemilum inescence method		2025-03-19
116	Cerebrospinal Fluid Protein (PROT)	CSF	pyrogallol red		2025-03-19
117	Carbamazepine	Serum	ChemiLuminescence		2025-03-19
118	Cystatin C	Serum	Immunoturbidimetry		2025-03-19
AD Clinical Immunology					

CHINA NATIONAL ACCREDITATION SERVICE FOR CONFORMITY ASSESSMENT
SCHEDULE OF ACCREDITATION CERTIFICATE
中国合格评定国家认可委员会
认可证书专用章
在线扫码获取验证

The scope of the accreditation in Chinese remains the definitive version.

CHINA NATIONAL ACCREDITATION SERVICE FOR CONFORMITY ASSESSMENT
SCHEDULE OF ACCREDITATION CERTIFICATE

№	Examination Item	Sample Type	Analytical Method	Note	Effective Date
119	Complement 3	Serum	Immunonephelometry		2025-03-19
120	Complement 4	Serum	Immunonephelometry		2025-03-19
121	Immunoglobulin G	Serum	Immunonephelometry		2025-03-19
122	Immunoglobulin M	Serum	Immunonephelometry		2025-03-19
123		Serum	Electrochemiluminescence immunoassay		2025-03-19
124	Immunoglobulin A	Serum	Immunonephelometry		2025-03-19
125	Light chain kappa	Serum	Immunonephelometry		2025-03-19
126	Light chain lambda		Immunonephelometry		2025-03-19
127			Immunoturbidimetry<br>Immunoturbidimetry		2025-03-19
128	Antinuclear antibody（ANA）	Serum	indirect immunofluorescence		2025-03-19
129	Anti-double-stranded DNA antibody（anti-dsDNA）	Serum	indirect immunofluorescence<br>Euroimmun immunity blotting		2025-03-19
130	Anti-Smith antibody（anti-Sm）	Serum	Euroimmun immunity blotting		2025-03-19

中国合格评定国家认可委员会 认可证书专用章

在线扫码获取验证

The scope of the accreditation in Chinese remains the definitive version.

CHINA NATIONAL ACCREDITATION SERVICE FOR CONFORMITY ASSESSMENT
SCHEDULE OF ACCREDITATION CERTIFICATE

№	Examination Item	Sample Type	Analytical Method	Note	Effective Date
131	Anti-nuclear ribonucleoprotein /Smith antibody（anti-nRNP/Sm）	Serum	Euroimmun immunity blotting		2025-03-19
132	Anti-sjögren syndrome A antigen antibody（anti-SS-A）	Serum	Euroimmun immunity blotting		2025-03-19
133	Anti-sjögren syndrome B antigen antibody（anti-SS-B）	Serum	Euroimmun immunity blotting		2025-03-19
134	Anti-scleroderma-70 antibody（anti-Scl-70）	Serum	Euroimmun immunity blotting		2025-03-19
135	Anti-John-1 antibody（anti-Jo-1）	Serum	Euroimmun immunity blotting		2025-03-19
136	Rheumatoid factor	Serum	Immunoneturbidimetric		2025-03-19
		Serum	Immunoneturbidimetric		2025-03-19
137	Interleukin 6（IL-6）	plasma	Electrochemistry luminescence method		2025-03-19
		plasma	Electrochemistry luminescence method		2025-03-19
138	HAV-IgM	Serum	Enzyme immunochemiluminescence		2025-03-19
139	Hepatitis B virus surface antigen（HBsAg）	Serum	Electrochemiluminescence immunoassay		2025-03-19
		Serum	Enzyme-linked immunoassay		2025-03-19
140	Hepatitis B virus surface antibody（HBsAb）	Serum	Electrochemiluminescence immunoassay		2025-03-19

中国合格评定国家认可委员会
认可证书专用章

The scope of the accreditation in Chinese remains the definitive version.

№	Examination Item	Sample Type	Analytical Method	Note	Effective Date
			Enzyme-linked immunoassay		
141	Hepatitis B virus e antigen （HBeAg）	Serum	Electrochemiluminescence immunoassay<br>Enzyme-linked immunoassay		2025-03-19
142	Hepatitis B virus e IgG antibody （HBeAb-IgG）	Serum	Electrochemiluminescence immunoassay<br>Enzyme-linked immunoassay		2025-03-19
143	Hepatitis B virus core antibody （HBcAb）	Serum	Electrochemiluminescence immunoassay<br>Enzyme-linked immunoassay		2025-03-19
144	Hepatitis C virus antibody （HCV-Ab）	Serum	Electrochemiluminescence immunoassay<br>Enzyme-linked immunoassay		2025-03-19
145	HEV IgM	Serum	Magnetic particle chemiluminescence		2025-03-19
146	HEV IgG	Serum	Magnetic particle chemiluminescence		2025-03-19
147	Human Immunodeficiency Virus antibody （HIV-Ab）	Serum	Enzyme-linked immunoassay		2025-03-19
148	Human Immunodeficiency Virus antigen/ antibody （HIV-Ag/Ab）	Serum	Electrochemiluminescence immunoassay		2025-03-19

CHINA NATIONAL ACCREDITATION SERVICE FOR CONFORMITY ASSESSMENT
SCHEDULE OF ACCREDITATION CERTIFICATE
中国合格评定国家认可委员会 认可证书专用章
在线扫码获取验证

The scope of the accreditation in Chinese remains the definitive version.

№	Examination Item	Sample Type	Analytical Method	Note	Effective Date
149	*T.pallidum*	Serum	Electrochemiluminescence immunoassay<br>agglutination		2025-03-19
150	*T.pallidum* non-specific antibody	Serum	agglutination		2025-03-19
151	Toxoplasma gondii IgM antibody	Serum	Electrochemiluminescence		2025-03-19
152	Anti Toxoplasma antibody IgG	Serum	Electrochemiluminescence		2025-03-19
153	RV-IgM	Serum	Electrochemiluminescence		2025-03-19
154	Anti rubella virus antibody IgG	Serum	Electrochemiluminescence		2025-03-19
155	Anti cytomegalovirus antibody IgM	Serum	Electrochemiluminescence		2025-03-19
156	Anti cytomegalovirus antibody IgG	Serum	Electrochemiluminescence		2025-03-19
157	HSV-Ⅰ IgM	Serum	Magnetic particle chemiluminescence		2025-03-19
158	HSV-Ⅰ IgG		Electrochemiluminescence		2025-03-19
159	HSV-Ⅱ IgM	Serum	Magnetic particle chemiluminescence		2025-03-19
160	HSV-Ⅱ IgG	Serum	Electrochemiluminescence		2025-03-19
161	Antistreptolysin O	Serum	Immunoneturbidimetric		2025-03-19

CHINA NATIONAL ACCREDITATION SERVICE FOR CONFORMITY ASSESSMENT
SCHEDULE OF ACCREDITATION CERTIFICATE
中国合格评定国家认可委员会 认可证书专用章
在线扫码获取验证

The scope of the accreditation in Chinese remains the definitive version.

CHINA NATIONAL ACCREDITATION SERVICE FOR CONFORMITY ASSESSMENT
SCHEDULE OF ACCREDITATION CERTIFICATE

№	Examination Item	Sample Type	Analytical Method	Note	Effective Date
			Immunoneturbidimetric		
162	Carcinoembryonic antigen	Serum	Electrochemiluminescence		2025-03-19
163	Alpha- fetoprotein	Serum	Electrochemiluminescence		2025-03-19
164	Prostate specific antigen	Serum	Electrochemiluminescence		2025-03-19
165	free PSA	Serum	Electrochemiluminescence		2025-03-19
166	CA50		Enzyme immunochemiluminescence		2025-03-19
167	CarbohydRate antigen12-5	Serum	Electrochemiluminescence		2025-03-19
168	CarbohydRate antigen15-3	Serum	Electrochemiluminescence		2025-03-19
169	CarbohydRate antigen19-9	Serum	Electrochemiluminescence		2025-03-19
170	CA242	Serum	Chemiluminescence immunoassay		2025-03-19
171	Pepsinogen I	Serum	Chemiluminescence immunoassay		2025-03-19
172	Pepsinogen II	Serum	Chemiluminescence immunoassay		2025-03-19
173	Anti-circum citrulline peptide antibody（ anti-CCP）	Serum	Chemiluminescent microparticle immunoassay		2025-03-19
AE Clinical microbiology					

中国合格评定国家认可委员会 认可证书专用章

在线扫码获取验证

The scope of the accreditation in Chinese remains the definitive version.

CHINA NATIONAL ACCREDITATION SERVICE FOR CONFORMITY ASSESSMENT
SCHEDULE OF ACCREDITATION CERTIFICATE

№	Examination Item	Sample Type	Analytical Method	Note	Effective Date
174	Gram staining direct smear microscopy for bacteria(specimens that is not need to concentrate)	sputum	Microscopy		2025-03-19
		pus	Microscopy		2025-03-19
		whole blood	Microscopy		2025-03-19
175	Gram staining concentrated smear microscopy for fungus (specimens that is need to concentrate)	Urine	Microscopy		2025-03-19
		pleural fluid	Microscopy		2025-03-19
		ascites fluids	Microscopy		2025-03-19
		puncture fluid	Microscopy		2025-03-19
		cerebrospinal fluid	Microscopy		2025-03-19
176	Acid fast staining direct smear microscopy（specimens that is not need to concentrate）	sputum	Microscopy		2025-03-19
		pus	Microscopy		2025-03-19
177	Acid fast staining concentrated smear microscopy （specimens that is need to concentrate）	Urine	Acid-fast staning		2025-03-19
		pleural fluid	Microscopy		2025-03-19
		ascites fluids	Microscopy		2025-03-19
		puncture fluid	Microscopy		2025-03-19

在线扫码获取验证

The scope of the accreditation in Chinese remains the definitive version.

№	Examination Item	Sample Type	Analytical Method	Note	Effective Date
		cerebrospinal fluid	Microscopy		2025-03-19
178	Modified acid fast staining smear microscopy for Nocardia	sputum	Microscopy		2025-03-19
		bronchoalveolar lavage fluid	Microscopy		2025-03-19

CHINA NATIONAL ACCREDITATION SERVICE FOR CONFORMITY ASSESSMENT
SCHEDULE OF ACCREDITATION CERTIFICATE

中国合格评定国家认可委员会
认可证书专用章

The scope of the accreditation in Chinese remains the definitive version.

№	Examination Item	Sample Type	Analytical Method	Note	Effective Date
179	Bacterial culture and identification	whole blood	Instruments method	Limited to Staphylococcus spp., Streptococcus spp. (except Streptococcus pyogenes, Streptococcus pneumoniae), Enterococcus spp., Escherichia spp., Enterobacter spp., Serratia spp., Klebsiella spp., Proteus spp., Citrobacter spp., Puluoweideng spp., Salmonella spp.,Pseudomonas spp., Acinetobacter spp., Alcaligenes, Bokehuode spp., Stenotrophomonas spp., Aeromonas spp., Moraxella spp., Pantoea spp., Corynebacterium spp., Listeria spp., Erysipelothrix spp., Rhodococcus spp., Arcanobacterium spp.	2025-03-19

CHINA NATIONAL ACCREDITATION SERVICE FOR CONFORMITY ASSESSMENT
SCHEDULE OF ACCREDITATION CERTIFICATE
合格评定国家认可委员会 认可证书专用章
在线扫码获取验证

The scope of the accreditation in Chinese remains the definitive version.

№	Examination Item	Sample Type	Analytical Method	Note	Effective Date
		pleural fluid	Instruments method	Limited to Staphylococcus spp., Streptococcus spp. (except Streptococcus pyogenes, Streptococcus pneumoniae), Enterococcus spp., Escherichia spp., Enterobacter spp., Serratia spp., Klebsiella spp., Proteus spp., Citrobacter spp., Puluoweideng spp., Salmonella spp., Pseudomonas spp., Acinetobacter spp., Alcaligenes, Bokehuode spp., Stenotrophomonas spp., Aeromonas spp., Moraxella spp., Pantoea spp., Corynebacterium spp., Listeria spp., Erysipelothrix spp., Rhodococcus spp., Arcanobacterium spp.	2025-03-19

CHINA NATIONAL ACCREDITATION SERVICE FOR CONFORMITY ASSESSMENT
SCHEDULE OF ACCREDITATION CERTIFICATE
中国合格评定国家认可委员会 认可证书专用章
在线扫码获取验证

The scope of the accreditation in Chinese remains the definitive version.

№	Examination Item	Sample Type	Analytical Method	Note	Effective Date
		ascites fluids	Instruments method	Limited to Staphylococcus spp., Streptococcus spp. (except Streptococcus pyogenes, Streptococcus pneumoniae), Enterococcus spp., Escherichia spp., Enterobacter spp., Serratia spp., Klebsiella spp., Proteus spp., Citrobacter spp., Puluoweideng spp., Salmonella spp., Pseudomonas spp., Acinetobacter spp., Alcaligenes, Bokehuode spp., Stenotrophomonas spp., Aeromonas spp., Moraxella spp., Pantoea spp., Corynebacterium spp., Listeria spp., Erysipelothrix spp., Rhodococcus spp., Arcanobacterium spp.	2025-03-19

CHINA NATIONAL ACCREDITATION SERVICE FOR CONFORMITY ASSESSMENT
SCHEDULE OF ACCREDITATION CERTIFICATE

中国合格评定国家认可委员会 认可证书专用章

The scope of the accreditation in Chinese remains the definitive version.

№	Examination Item	Sample Type	Analytical Method	Note	Effective Date
		cerebrospinal fluid	Instruments method	Limited to Staphylococcus spp., Streptococcus spp. (except Streptococcus pyogenes, Streptococcus pneumoniae), Enterococcus spp., Escherichia spp., Enterobacter spp., Serratia spp., Klebsiella spp., Proteus spp., Citrobacter spp., Puluoweideng spp., Salmonella spp., Pseudomonas spp., Acinetobacter spp., Alcaligenes, Bokehuode spp., Stenotrophomonas spp., Aeromonas spp., Moraxella spp., Pantoea spp., Corynebacterium spp., Listeria spp., Erysipelothrix spp., Rhodococcus spp., Arcanobacterium spp.	2025-03-19

CHINA NATIONAL ACCREDITATION SERVICE FOR CONFORMITY ASSESSMENT
SCHEDULE OF ACCREDITATION CERTIFICATE

中国合格评定国家认可委员会
认可证书专用章

The scope of the accreditation in Chinese remains the definitive version.

№	Examination Item	Sample Type	Analytical Method	Note	Effective Date
		puncture fluid	Instruments method	Limited to Staphylococcus spp., Streptococcus spp. (except Streptococcus pyogenes, Streptococcus pneumoniae), Enterococcus spp., Escherichia spp., Enterobacter spp., Serratia spp., Klebsiella spp., Proteus spp., Citrobacter spp., Puluoweideng spp., Salmonella spp., Pseudomonas spp., Acinetobacter spp., Alcaligenes, Bokehuode spp., Stenotrophomonas spp., Aeromonas spp., Moraxella spp., Pantoea spp., Corynebacterium spp., Listeria spp., Erysipelothrix spp., Rhodococcus spp., Arcanobacterium spp.	2025-03-19

CHINA NATIONAL ACCREDITATION SERVICE FOR CONFORMITY ASSESSMENT
SCHEDULE OF ACCREDITATION CERTIFICATE

中国合格评定国家认可委员会 认可证书专用章

在线扫码获取验证

The scope of the accreditation in Chinese remains the definitive version.

CHINA NATIONAL ACCREDITATION SERVICE FOR CONFORMITY ASSESSMENT
SCHEDULE OF ACCREDITATION CERTIFICATE

№	Examination Item	Sample Type	Analytical Method	Note	Effective Date
		urine	Instruments method	Limited to Staphylococcus spp., Streptococcus spp. (except Streptococcus pyogenes, Streptococcus pneumoniae), Enterococcus spp., Escherichia spp., Enterobacter spp., Serratia spp., Klebsiella spp., Proteus spp., Citrobacter spp., Puluoweideng spp., Salmonella spp., Pseudomonas spp., Acinetobacter spp., Alcaligenes, Bokehuode spp., Stenotrophomonas spp., Aeromonas spp., Moraxella spp., Pantoea spp., Corynebacterium spp., Listeria spp., Erysipelothrix spp., Rhodococcus spp., Arcanobacterium spp.	2025-03-19
		stool	Instruments method	Limited to Salmonella spp, Shigella spp.	2025-03-19

合格评定国家认可委员会 认可证书专用章

The scope of the accreditation in Chinese remains the definitive version.

CHINA NATIONAL ACCREDITATION SERVICE FOR CONFORMITY ASSESSMENT
SCHEDULE OF ACCREDITATION CERTIFICATE

№	Examination Item	Sample Type	Analytical Method	Note	Effective Date
		pus	Instruments method	Limited to Staphylococcus spp., Streptococcus spp. (except Streptococcus pyogenes, Streptococcus pneumoniae), Enterococcus spp., Escherichia spp., Enterobacter spp., Serratia spp., Klebsiella spp., Proteus spp., Citrobacter spp., Puluoweideng spp., Salmonella spp., Pseudomonas spp., Acinetobacter spp., Alcaligenes, Bokehuode spp., Stenotrophomonas spp., Aeromonas spp., Moraxella spp., Pantoea spp., Corynebacterium spp., Listeria spp., Erysipelothrix spp., Rhodococcus spp., Arcanobacterium spp.	2025-03-19

合格评定国家认可委员会 认可证书专用章

The scope of the accreditation in Chinese remains the definitive version.

CHINA NATIONAL ACCREDITATION SERVICE FOR CONFORMITY ASSESSMENT
SCHEDULE OF ACCREDITATION CERTIFICATE

№	Examination Item	Sample Type	Analytical Method	Note	Effective Date
		sputum	Instruments method	Limited to Staphylococcus spp., Streptococcus spp. (except Streptococcus pyogenes, Streptococcus pneumoniae), Enterococcus spp., Escherichia spp., Enterobacter spp., Serratia spp., Klebsiella spp., Proteus spp., Citrobacter spp., Puluoweideng spp., Salmonella spp., Pseudomonas spp., Acinetobacter spp., Alcaligenes, Bokehuode spp., Stenotrophomonas spp., Aeromonas spp., Moraxella spp., Pantoea spp., Corynebacterium spp., Listeria spp., Erysipelothrix spp., Rhodococcus spp., Arcanobacterium spp.	2025-03-19

合格评定国家认可委员会 认可证书专用章

The scope of the accreditation in Chinese remains the definitive version.

CHINA NATIONAL ACCREDITATION SERVICE FOR CONFORMITY ASSESSMENT
SCHEDULE OF ACCREDITATION CERTIFICATE

№	Examination Item	Sample Type	Analytical Method	Note	Effective Date
		bronchoalveolar lavage fluid	Instruments method	Limited to Staphylococcus spp., Streptococcus spp. (except Streptococcus pyogenes, Streptococcus pneumoniae), Enterococcus spp., Escherichia spp., Enterobacter spp., Serratia spp., Klebsiella spp., Proteus spp., Citrobacter spp., Puluoweideng spp., Salmonella spp., Pseudomonas spp., Acinetobacter spp., Alcaligenes, Bokehuode spp., Stenotrophomonas spp., Aeromonas spp., Moraxella spp., Pantoea spp., Corynebacterium spp., Listeria spp., Erysipelothrix spp., Rhodococcus spp., Arcanobacterium spp.	2025-03-19

在线扫码获取验证

The scope of the accreditation in Chinese remains the definitive version.

№	Examination Item	Sample Type	Analytical Method	Note	Effective Date
		pharynged swab	Instruments method	Limited to Staphylococcus spp., Streptococcus spp. (except Streptococcus pyogenes, Streptococcus pneumoniae), Enterococcus spp., Escherichia spp., Enterobacter spp., Serratia spp., Klebsiella spp., Proteus spp., Citrobacter spp., Puluoweideng spp., Salmonella spp., Pseudomonas spp., Acinetobacter spp., Alcaligenes, Bokehuode spp., Stenotrophomonas spp., Aeromonas spp., Moraxella spp., Pantoea spp., Corynebacterium spp., Listeria spp., Erysipelothrix spp., Rhodococcus spp., Arcanobacterium spp.	2025-03-19

CHINA NATIONAL ACCREDITATION SERVICE FOR CONFORMITY ASSESSMENT
SCHEDULE OF ACCREDITATION CERTIFICATE

中国合格评定国家认可委员会 认可证书专用章

在线扫码获取验证

The scope of the accreditation in Chinese remains the definitive version.

CHINA NATIONAL ACCREDITATION SERVICE FOR CONFORMITY ASSESSMENT
SCHEDULE OF ACCREDITATION CERTIFICATE

№	Examination Item	Sample Type	Analytical Method	Note	Effective Date
		wound swab	Instruments method	Limited to Staphylococcus spp., Streptococcus spp. (except Streptococcus pyogenes, Streptococcus pneumoniae), Enterococcus spp., Escherichia spp., Enterobacter spp., Serratia spp., Klebsiella spp., Proteus spp., Citrobacter spp., Puluoweideng spp., Salmonella spp., Pseudomonas spp., Acinetobacter spp., Alcaligenes, Bokehuode spp., Stenotrophomonas spp., Aeromonas spp., Moraxella spp., Pantoea spp., Corynebacterium spp., Listeria spp., Erysipelothrix spp., Rhodococcus spp., Arcanobacterium spp.	2025-03-19
180	Bacterial culture and identification，Streptococcus pyogenes	pharyngeal swab	Instruments method		2025-03-19

在线扫码获取验证

The scope of the accreditation in Chinese remains the definitive version.

CHINA NATIONAL ACCREDITATION SERVICE FOR CONFORMITY ASSESSMENT
SCHEDULE OF ACCREDITATION CERTIFICATE

№	Examination Item	Sample Type	Analytical Method	Note	Effective Date
		sputum	Instruments method		2025-03-19
		bronchoalveolar lavage fluid	Instruments method		2025-03-19
		pus	Instruments method		2025-03-19
		wound swab	Instruments method		2025-03-19
		whole blood	Instruments method		2025-03-19
181	Bacterial culture and identification,Streptococcus,group B	Cervical secretions	Instruments method		2025-03-19
		urine	Instruments method		2025-03-19
		pus	Instruments method		2025-03-19
		whole blood	Instruments method		2025-03-19
182	Bacterial culture and identification，Haemophilus influenzae	sputum	Instruments method		2025-03-19
		pharyngeal swab	Instruments method		2025-03-19
		bronchoalveolar lavage fluid	Instruments method		2025-03-19
		whole blood	Instruments method		2025-03-19
183	Bacterial culture and identification，Streptococcus	sputum	Instruments method		2025-03-19

中国合格评定国家认可委员会
认可证书专用章

在线扫码获取验证

The scope of the accreditation in Chinese remains the definitive version.

CHINA NATIONAL ACCREDITATION SERVICE FOR CONFORMITY ASSESSMENT
SCHEDULE OF ACCREDITATION CERTIFICATE

№	Examination Item	Sample Type	Analytical Method	Note	Effective Date
	pneumoniae	pharyngeal swab	Instruments method		2025-03-19
		bronchoalveolar lavage fluid	Instruments method		2025-03-19
		whole blood	Instruments method		2025-03-19
184	Bacterial culture and identification，Neisseria meningitidis	Sputum	Instruments method		2025-03-19
		cerebrospinal fluid	Instruments method		2025-03-19
185	Antimicrobial susceptibility qualitative test，aerobes	strains	Kirby-Bauer method	Limited to bacterial genera / species with judgment criteria in CLSIM100 or CLSIM45 files	2025-03-19
186	Antimicrobial susceptibility quantitative test，aerobes	strains	Instruments method		2025-03-19
187	Serotyping，Salmonella spp.	strains	agglutination test		2025-03-19
188	Serotyping，Shigella spp.	strains	agglutination test		2025-03-19
189	Serotyping，Vibrio cholera	strains	agglutination test		2025-03-19
190	Bacterial culture and identification，Vibrio cholera	stool	Instruments method		2025-03-19
191	Gram staining direct smear microscopy for fungus（specimens that is not need to concentrate）	sputum	Microscopy		2025-03-19

The scope of the accreditation in Chinese remains the definitive version.

CHINA NATIONAL ACCREDITATION SERVICE FOR CONFORMITY ASSESSMENT
SCHEDULE OF ACCREDITATION CERTIFICATE

№	Examination Item	Sample Type	Analytical Method	Note	Effective Date
		pus	Microscopy		2025-03-19
		whole blood	Microscopy		2025-03-19
192	Gram staining concentrated smear microscopy for fungus（specimens that is need to concentrate）	urine	Microscopy		2025-03-19
		pleural fluid	Microscopy		2025-03-19
		ascites fluids	Microscopy		2025-03-19
		puncture fluid	Microscopy		2025-03-19
		cerebrospinal fluid	Microscopy		2025-03-19
193	Ink stain	Cerebrospinal fluid	Microscopy		2025-03-19
194	Candida culture and identification	sputum	Instruments method		2025-03-19
		bronchoalveolar lavage fluid	Instruments method		2025-03-19
		urine	Instruments method		2025-03-19
		pus	Instruments method		2025-03-19
		wound swab	Instruments method		2025-03-19
		whole blood	Instruments method		2025-03-19

中国合格评定国家认可委员会
认可证书专用章
在线扫码获取验证

The scope of the accreditation in Chinese remains the definitive version.

CHINA NATIONAL ACCREDITATION SERVICE FOR CONFORMITY ASSESSMENT
SCHEDULE OF ACCREDITATION CERTIFICATE

№	Examination Item	Sample Type	Analytical Method	Note	Effective Date
		cerebrospinal fluid	Instruments method		2025-03-19
		pleural fluid	Instruments method		2025-03-19
		ascites fluid	Instruments method		2025-03-19
		puncture fluid	Instruments method		2025-03-19
B Transfusion medicine					
BA Blood group of red cells					
195	ABO blood group forward typing	erythrocyte	microcolumn agglutination method<br>microcolumn agglutination method<br>microcolumn agglutination method(manual）		2025-03-19
196	ABO blood group reverse typing	plasma/ serum	microcolumn agglutination method<br>microcolumn agglutination method<br>microcolumn agglutination method(manual）		2025-03-19
197	RhD blood group (RhD antigen)	erythrocyte	microcolumn agglutination method<br>microcolumn agglutination method		2025-03-19

中国合格评定国家认可委员会
认可证书专用章

在线扫码获取验证

The scope of the accreditation in Chinese remains the definitive version.

№	Examination Item	Sample Type	Analytical Method	Note	Effective Date
			microcolumn agglutination method(manual）		
198	Irregular antibody screening	plasma/ serum	microcolumn agglutination method		2025-03-19
			microcolumn agglutination method(manual）		
199	Crossmatching	whole blood	polybrene method		2025-03-19
			microcolumn agglutination method(manual）		
X Molecular diagnosis					
XA Molecular test of pathogen					
200	Hepatitis B virus desoxyribonucleic acid（HBV DNA）	Serum	Real-time PCR		2025-03-19
			Real-time PCR(internal standard method)		
201	Hepatitis C virus ribonucleic acid	Plasma	Real-time PCR		2025-03-19
202	Cytomegalovirus desoxyribonucleic acid（CMV DNA）	Whole blood	Real-time PCR		2025-03-19
203	EB virus ribonucleic acid（EB-DNA）	Whole blood	Real-time PCR		2025-03-19
204	Human papilloma virus desoxyribonucleic acid（HPV DNA）	Exfoliated cells of Gynecology	Real-time PCR		2025-03-19

CHINA NATIONAL ACCREDITATION SERVICE FOR CONFORMITY ASSESSMENT
SCHEDULE OF ACCREDITATION CERTIFICATE

在线扫码获取验证

The scope of the accreditation in Chinese remains the definitive version.

№	Examination Item	Sample Type	Analytical Method	Note	Effective Date
205	Human papilloma virus genotyping	Exfoliated cells of Gynecology	Real-time PCR		2025-03-19
206	TB ribonucleic acid(TB-DNA)	sputum	Real-time PCR		2025-03-19
207	Neisseria gonorrhoeae ribonucleic acid	Urine/Secretion	simultaneous amplification and testing(SAT)		2025-03-19
208	Chlamydia trachomatis ribonucleic acid	Urine/Secretion	simultaneous amplification and testing(SAT)		2025-03-19
209	Ureaplasma Urealyticum ribonucleic acid	Urine/Secretion	simultaneous amplification and testing(SAT)		2025-03-19
210	mycoplasma genitalium ribonucleic acid	Urine/Secretion	simultaneous amplification and testing(SAT)		2025-03-19
XB Molecular test of genetic disease and cytogenetic analysis					
211	α -Thalassaemia gene mutations	Whole blood	gap-PCR		2025-03-19
212	β -Thalassaemia gene mutations	Whole blood	PCR-reverse dot hybridization		2025-03-19
213	chromosome karyotyping	Whole Blood	Giemsa banding		2025-03-19
Y Flow Cytometry					
YB Other Disorders					
214	Tatal T lymphocyte count （TLC）	Whole blood	Flow cytometry		2025-03-19
215	T lyophocyte subsets	Whole blood	Flow cytometry		2025-03-19

CHINA NATIONAL ACCREDITATION SERVICE FOR CONFORMITY ASSESSMENT
SCHEDULE OF ACCREDITATION CERTIFICATE
中国合格评定国家认可委员会 认可证书专用章
在线扫码获取验证

The scope of the accreditation in Chinese remains the definitive version.

№	Examination Item	Sample Type	Analytical Method	Note	Effective Date
216	B lymphocyte count	Whole blood	Flow cytometry		2025-03-19
217	Nature killer cell count（NK）	Whole blood	Flow cytometry		2025-03-19
218	HLA-B27	Whole blood	Flow cytometry		2025-03-19

CHINA NATIONAL ACCREDITATION SERVICE FOR CONFORMITY ASSESSMENT
SCHEDULE OF ACCREDITATION CERTIFICATE

中国合格评定国家认可委员会
认可证书专用章

The scope of the accreditation in Chinese remains the definitive version.
