# Supplementary material for: Diagnostic accuracy of AMH for primary ovarian insufficiency/premature ovarian failure: a real-world cohort study
Source: Front Endocrinol (Lausanne). 2026 Feb 11;17:1742145. doi: 10.3389/fendo.2026.1742145 (PMC12932242; doi:10.3389/fendo.2026.1742145)
Supplement: Supplementary file 1 [file DataSheet1.zip › Quality Control Certificates/广东省中医院检验科ISO证书2013-2016 英文.pdf]

CHINA NATIONAL ACCREDITATION SERVICE FOR CONFORMITY ASSESSMENT

APPENDIX OF LABORATORY ACCREDITATION CERTIFICATE

(Registration No. CNAS MT0003)

NAME:Clinical Laboratory of Guangdong Provincial Hospital of Traditional Chinese Medicine

ADDRESS:No.111, Dade Road, Guangzhou, Guangdong, China

Accreditation Criteria: ISO 15189:2007 and relevant requirements of CNAS

Date of Issue:2013-12-19 **Date of Expiry:2016-12-18**

Date of Update:2013-12-19

APPENDIX 2 ACCREDITED EXAMINATION

№	Examination Item	Sample Type	Code	Analytical Method	Notes
1	Alanine aminotransferase	Serum	3B045-0000-022-272-01	Rate	
		Serum	3B045-0000-022-272-01	Rate	
		Serum	3B045-0000-022-272-01	Dry Chemistry	
2	Aspartate aminotransferase	Serum	3B035-0000-022-272-01	Rate	
		Serum	3B035-0000-022-272-01	Rate	
		Serum	3B035-0000-022-272-01	Dry Chemistry	
3	Total protein	Serum	3A010-0000-022-271-01	biuret method	
		Serum	3A010-0000-022-271-01	biuret method	
		Serum	3A010-0000-022-271-01	Dry Chemistry	
4	Albumin	Serum	3B015-7052-022-271-01	bromcresol green method(BCG)	

Should there be any inconsistencies between Chinese and English versions of the scope of accreditation, the Chinese version shall prevail in that the English version is provided by the conformity assessment body and is for reference only.

№	Examination Item	Sample Type	Code	Analytical Method	Notes
35	Cerebrospinal Fluid Protein (PROT)	CSF	1C010-0000-023-291-01	Dry Chemistry	
36	Amylase	Serum	3B160-0000-022-291-01	EPS-G7	
		Serum	3B160-0000-022-291-01	EPS-G7	
		Serum	3B160-0000-022-291-01	Dry Chemistry	
37	Sodium	Serum	3H010-0000-022-261-01	ISE-indirect	
		Serum	3H010-0000-022-261-01	ISE-indirect	
		Serum	3H010-0000-022-261-01	Dry Chemistry	
38	Potassium	Serum	3H015-0000-022-261-01	ISE-indirect	
		Serum	3H015-0000-022-261-01	ISE-indirect	
		Serum	3H015-0000-022-291-01	Dry Chemistry	
39	Chloride	Serum	3H020-0000-022-261-01	ISE-indirect	
		Serum	3H020-0000-022-261-01	ISE-indirect	
		Serum	3H020-0000-022-261-01	Dry Chemistry	
40	Follicle-stimulating hormone	Serum	4A010-8216-023-051-01	ChemiLuminescence	
41	Luteinizing hormone	Serum	4A009-8210-023-051-01	ChemiLuminescence	
42	Estradiol	Serum	4F004-8418-023-051-01	ChemiLuminescence	
43	Testosterone	Serum	4F014-8426-023-051-01	ChemiLuminescence	
44	Prolactin	Serum	4A007-8224-023-051-01	ChemiLuminescence	
45	Progesterone	Serum	4F008-8424-023-051-01	ChemiLuminescence	
46	Triiodothyronine	Serum	4B001-0000-023-051-01	ChemiLuminescence	

Should there be any inconsistencies between Chinese and English versions of the scope of accreditation, the Chinese version shall prevail in that the English version is provided by the conformity assessment body and is for reference only.

№	Examination Item	Sample Type	Code	Analytical Method	Notes
98	Treponema pallidum particle agglutination	serum	5E073-0000-023-103-11	agglutination	
99	pH	whole blood	3H050-0000-018-261-01	Electrode-direct	
		whole blood	3H050-0000-018-261-01	Electrode-direct	
100	Partial pressure of oxygen	whole blood	3H060-0000-018-261-01	Electrode-direct	
		whole blood	3H060-0000-018-261-01	Electrode-direct	
101	Partial pressure of carbon dioxide	whole blood	3H055-0000-018-261-01	Electrode-direct	
		whole blood	3H055-0000-018-261-01	Electrode-direct	
102	Prothrombin time	plasma	2B030135202231103	clotting time method	
		plasma	2B030135202231103	clotting time method	
103	International Normalized Ratio	plasma	2B030000002291901	calculation	
		plasma	2B030000002291901	calculation	
104	Fibrinogen	plasma	2B100135202231101	clotting time method	
		plasma	2B100135202231101	clotting time method	
105	Activated partial thromboplastin time	plasma	2B020135202231103	clotting time method	
		plasma	2B020135202231103	clotting time method	
106	White blood cell count	Whole blood	2A010135201930101	Laser scattering/nucleic acid fluorescence	
		Whole blood	2A010135201930101	Laser scattering/nucleic acid fluorescence	

www.docin.com

Should there be any inconsistencies between Chinese and English versions of the scope of accreditation, the Chinese version shall prevail in that the English version is provided by the conformity assessment body and is for reference only.

№	Examination Item	Sample Type	Code	Analytical Method	Notes
106	White blood cell count	Whole blood	2A0101352019 30101	Laser scattering/nucleic acid fluorescence	
		Whole blood	2A0101352019 30101	Laser scattering/nucleic acid fluorescence	
107	Red blood cell count	Whole blood	2A0101352019 30101	impedance technology	
	Red blood cell count	Whole blood	2A0101352019 30101	impedance technology	
	Red blood cell count	Whole blood	2A0101352019 30101	impedance technology	
	Red blood cell count	Whole blood	2A0101352019 30101	impedance technology	
108	Platelet count	Whole blood	2A0101352019 30101	impedance technology	
	Platelet count	Whole blood	2A0101352019 30101	impedance technology	
	Platelet count	Whole blood	2A0101352019 30101	impedance technology	
	Platelet count	Whole blood	2A0101352019 30101	impedance technology	
109	Hemoglobin	Whole blood	2A0101352019 27601	visible absorption spectrophotometry	
	Hemoglobin	Whole blood	2A0101352019 27601	visible absorption spectrophotometry	
	Hemoglobin	Whole blood	2A0101352019 27601	visible absorption spectrophotometry	
	Hemoglobin	Whole blood	2A0101352019 27601	visible absorption spectrophotometry	
110	Hematocrit	Whole blood	2A0101352019 30101	impedance technology	
	Hematocrit	Whole blood	2A0101352019 30101	impedance technology	
	Hematocrit	Whole blood	2A0101352019 30101	impedance technology	

Should there be any inconsistencies between Chinese and English versions of the scope of accreditation, the Chinese version shall prevail in that the English version is provided by the conformity assessment body and is for reference only.

www.docin.com

№	Examination Item	Sample Type	Code	Analytical Method	Notes
110	Hematocrit	Whole blood	2A0101352019 30101	impedance technology	
111	Mean corpuscular volume	Whole blood	2A0101352019 30101	impedance technology	
	Mean corpuscular volume	Whole blood	2A0101352019 30101	impedance technology	
	Mean corpuscular volume	Whole blood	2A0101352019 30101	impedance technology	
	Mean corpuscular volume	Whole blood	2A0101352019 30101	impedance technology	
112	Mean corpusclar hemoglobin	Whole blood	2A0801352019 91901	calculation	
	Mean corpusclar hemoglobin	Whole blood	2A0801352019 91901	calculation	
	Mean corpusclar hemoglobin	Whole blood	2A0801352019 91901	calculation	
	Mean corpusclar hemoglobin	Whole blood	2A0801352019 91901	calculation	
113	Mean corpusclar hemoglobin concentration	Whole blood	2A0801352019 91901	calculation	
	Mean corpusclar hemoglobin concentration	Whole blood	2A0801352019 91901	calculation	
	Mean corpusclar hemoglobin concentration	Whole blood	2A0801352019 91901	calculation	
	Mean corpusclar hemoglobin concentration	Whole blood	2A0801352019 91901	calculation	
114	Leukocyte differential count	Whole blood	2A1601351019 30101	Laser scattering/nucleic acid fluorescence	

docin 豆丁 www.docin.com

Should there be any inconsistencies between Chinese and English versions of the scope of accreditation, the Chinese version shall prevail in that the English version is provided by the conformity assessment body and is for reference only.

№	Examination Item	Sample Type	Code	Analytical Method	Notes
114	Leukocyte differential count	Whole blood	2A160135101930101	Laser scattering/nucleic acid fluorescence	
	Leukocyte differential count	Whole blood	2A160135101930101	Laser scattering/nucleic acid fluorescence	
	Leukocyte differential count	Whole blood	2A160135101930101	Laser scattering/nucleic acid fluorescence	
115	Blood cell morphology	Whole blood	2A160135101960101	stain for blood cells	
	Blood cell morphology	Whole blood	2A160135101960101	stain for blood cells	
116	Erythrocyte sedimentation rate	Whole blood	2Z010135201909601	other precipitation methods	
117	ABO blood grouping identification	whole blood	5H010-1351-024-101-11	Column agglutination Method	
	ABO blood grouping identification	whole blood	5H010-1351-024-101-11	Column agglutination Method	
	ABO blood grouping identification	whole blood	5H010-1351-024-101-11	Tube Method	
118	Rh(D) blood grouping identification	whole blood	5H020-1351-024-101-11	Column agglutination Method	
	Rh(D) blood grouping identification	whole blood	5H020-1351-024-101-11		
	Rh(D) blood grouping identification	whole blood	5H020-1351-024-101-11	Tube Method	
119	Specific gravity	urine	1A030135200390112	color formation	
		urine	1A030135200390112	color formation	
		urine	1A030135200390112	color formation	
		urine	1A030135200390112	color formation	
120	Protein	urine	1A030135200390112	color formation	

Should there be any inconsistencies between Chinese and English versions of the scope of accreditation, the Chinese version shall prevail in that the English version is provided by the conformity assessment body and is for reference only.

№	Examination Item	Sample Type	Code	Analytical Method	Notes
120	Protein	urine	1A0301352003 90112	color formation	
		urine	1A0301352003 90112	color formation	
		urine	1A0301352003 90112	color formation	
121	Glucose	urine	1A0301352003 90112	color formation	
		urine	1A0301352003 90112	color formation	
		urine	1A0301352003 90112	color formation	
		urine	1A0301352003 90112	color formation	
122	Nitrite	urine	1A0301352003 90112	color formation	
		urine	1A0301352003 90112	color formation	
		urine	1A0301352003 90112	color formation	
		urine	1A0301352003 90112	color formation	
123	Bilirubin	urine	1A0301352003 90112	color formation	
		urine	1A0301352003 90112	color formation	
		urine	1A0301352003 90112	color formation	
		urine	1A0301352003 90112	color formation	
124	urobilinogen	urine	1A0301352003 90112	color formation	
		urine	1A0301352003 90112	color formation	
		urine	1A0301352003 90112	color formation	
		urine	1A0301352003 90112	color formation	
125	pH	urine	1A0301352003 90112	color formation	
		urine	1A0301352003 90112	color formation	
		urine	1A0301352003 90112	color formation	
		urine	1A0301352003 90112	color formation	

docin 豆丁 www.docin.com

Should there be any inconsistencies between Chinese and English versions of the scope of accreditation, the Chinese version shall prevail in that the English version is provided by the conformity assessment body and is for reference only.

№	Examination Item	Sample Type	Code	Analytical Method	Notes
126	Ketones	urine	1A0301352003 90112	color formation	
		urine	1A0301352003 90112	color formation	
		urine	1A0301352003 90112	color formation	
		urine	1A0301352003 90112	color formation	
127	Leucocyte eastase	urine	1A0301352003 90112	color formation	
		urine	1A0301352003 90112	color formation	
		urine	1A0301352003 90112	color formation	
		urine	1A0301352003 90112	color formation	
128	blood	urine	1A0301352003 90112	color formation	
		urine	1A0301352003 90112	color formation	
		urine	1A0301352003 90112	color formation	
		urine	1A0301352003 90112	color formation	
129	Urinary sediment	urine	1A1050000003 92013	microscope examination	
		urine	1A1050000003 70113	auto-identification of microscope image	
		urine	1A1050000003 70113	microscope examination	
		urine	1A1050000003 70113	microscope examination	
130	Occult blood	feces	1B0351351015 90111	other immunoassays	
131	Feces routine	feces	1B0351351015 90111	microscope examination	
		feces	1B0351351015 90111	microscope examination	

www.docin.com

Should there be any inconsistencies between Chinese and English versions of the scope of accreditation, the Chinese version shall prevail in that the English version is provided by the conformity assessment body and is for reference only.
